# Extending greedy feature selection algorithms to multiple solutions

**DOI:** 10.1007/s10618-020-00731-7

**Published:** 2021-05-01

**Authors:** Giorgos Borboudakis, Ioannis Tsamardinos

**Affiliations:** 1 University of Crete, Heraklion, Greece; 2 Gnosis Data Analysis (JADBio), Heraklion, Greece; 3 Institute of Applied and Computational Mathematics, Foundation for Research and Technology, Hellas, Greece

**Keywords:** Feature selection, Multiple solutions, Multiple feature selection, Stepwise selection

## Abstract

Most feature selection methods identify only a single solution. This is acceptable for predictive purposes, but is not sufficient for knowledge discovery if multiple solutions exist. We propose a strategy to extend a class of greedy methods to efficiently identify multiple solutions, and show under which conditions it identifies all solutions. We also introduce a taxonomy of features that takes the existence of multiple solutions into account. Furthermore, we explore different definitions of statistical equivalence of solutions, as well as methods for testing equivalence. A novel algorithm for compactly representing and visualizing multiple solutions is also introduced. In experiments we show that (a) the proposed algorithm is significantly more computationally efficient than the TIE* algorithm, the only alternative approach with similar theoretical guarantees, while identifying similar solutions to it, and (b) that the identified solutions have similar predictive performance.

## Introduction

Feature selection is an essential part of data analysis tasks which focus on knowledge discovery and improving understanding of the problem under study. This is no accident, as the solution has been shown to be directly related to the data-generating causal mechanism (Koller and Sahami 1996; Tsamardinos and Aliferis 2003; Aliferis et al. 2010). In many domains, such as molecular biology or life sciences, feature selection is often the main objective, not the resulting predictive model. Other advantages of feature selection are that it may improve predictive performance, especially in high-dimensional problems, and that it reduces the cost (monetary, computational, time and effort of measuring features) of making a model operational.

A shortcoming of existing methods is that they arbitrarily identify only one solution to the feature selection problem. There is growing evidence however, that in practice, multiple equivalent solutions often exist (Dougherty and Brun 2006; Roepman et al. 2006; Statnikov and Aliferis 2010; Statnikov et al. 2013; Karstoft et al. 2015). There are several reasons for their existence. In finite sample cases, the difference in predictive performance between two solutions may not be statistically distinguishable. In domains such as molecular biology there often exist multiple solutions, possibly because of the inherent redundancy present in the underlying biological system (Dougherty and Brun 2006; Statnikov and Aliferis 2010). In business applications, features are constructed in a way that may be deterministically related and thus could substitute one for the other in a solution. We argue that, while finding a single solution may be acceptable for building a predictive model, it is not sufficient when feature selection is employed for knowledge discovery. On the contrary, it may be misleading. For example, if several sets of risk factors in a medical study are collectively equally predictive of an event, then it is misleading to return only one of them and claim that the rest are superfluous. Indeed, they are given the selected ones, but it should be noted that there are other solutions that should also be considered. Ideally, a feature selection algorithm should identify all solutions that are “equivalent” (for some reasonable definition of equivalence). Another advantage of outputting multiple solutions is that one could use any of them for building a predictive model. This is especially important if measuring different features has different cost. The problem where each feature is associated with a cost is called *cost-sensitive feature selection* (He et al. 2012). Methods returning multiple solutions would deal with this by selecting the lowest-cost feature set among all equivalent solutions, while retaining the predictive ability of the model (Statnikov et al. 2013). Finally, it is important to note that the problem of multiple feature selection is also directly tied to the *stability of feature selection methods*. Stability is measured by how sensitive the output is when presented with different datasets sampled from the same distribution; see (Kalousis et al. 2007) for a study of stability of feature selection algorithms, and (Nogueira and Brown 2016) for methods of measuring stability. A single-solution feature selection algorithm is free to arbitrarily return any solution to the problem; when there are multiple solutions, the algorithm exhibits large instability during cross-validation. However, this is an artifact of how stability is measured: the algorithm is not to blame. Even an ideal single-solution algorithm would appear unstable in this case. When multiple solutions are present, measures of stability defined on single solutions become meaningless. We argue instead, that stability has to be defined in the context of multiple solutions. In this case, the ideal multiple-solutions algorithm should exhibit full stability asymptotically.

There exists only little work on the problem of identifying multiple solutions, and most existing algorithms are based on heuristic approaches with little to no theoretical guarantees. Examples include methods that repeatedly apply a feature selection algorithm with some element of randomness (e.g., by resampling the data) (Michiels et al. 2005; Peña et al. 2007), or methods that perform clustering to identify groups of highly correlated features (i.e., trying to identify groups of features that give similar predictive information) (Liu et al. 2010; Huang et al. 2014; Klasen et al. 2016); a more detailed review can be found in (Statnikov et al. 2013). Another class of algorithms includes causal-based methods that try to identify features that give the same information about the target in the context of other features (Tsamardinos et al. 2013; Statnikov et al. 2013; Lagani et al. 2017). Finally, the only algorithm that is able to provably identify all equivalent solutions (under certain conditions) is TIE* (Statnikov et al. 2013). Its drawbacks are that it is computationally demanding, and quite complex to understand and implement efficiently.

The main contributions of this work follow. In Sect. 3 we show that the taxonomy of features proposed by John et al. (1994) is counter-intuitive in the presence of multiple solutions, and propose an alternative taxonomy that takes multiple solutions into account. In Sect. 4 we state three definitions of statistical equivalence of feature sets, show how they are related, and make connections to the problem of model selection. Then, in Sect. 5 we propose a general template for feature selection algorithms that consist of a greedy forward phase and an optional backward phase. Examples of such methods are forward selection (Kutner et al. 2004; Weisberg 2005) and variations thereof (Margaritis and Thrun 2000; Margaritis 2009; Tsamardinos et al. 2003b), information-theoretic methods (Brown et al. 2012), as well as causal-based methods (Aliferis et al. 2010). In Sect. 6 we propose a simple and computationally efficient strategy to extend algorithms that fit into the proposed template for identifying multiple solutions and show that, under certain conditions, it identifies all equivalent solutions. In Sect. 7 we propose a method to compactly represent and visualize equivalent solutions, improving the interpretability of the results, which is especially useful if the number of equivalent solutions is high. Finally, in Sect. 8 we compare the proposed strategy to TIE* (Statnikov et al. 2013) and show that both methods produce similar results, with the proposed algorithm often being orders of magnitude faster.

## Preliminaries

We use upper-case letters for single variables, and bold upper-case letters for sets of variables. For an ordered set ${\mathbf {Z}}$, ${\mathbf {Z}}_{1:n}$ denotes the subset with the first *n* variables. The terms variable or feature will be used interchangeably. The set of all variables present in a dataset ${\mathcal {D}}$ except for *T* will be denoted as ${\mathbf {F}}$. The dataset obtained by excluding a set of variables ${\mathbf {E}}$ from ${\mathcal {D}}$ will be denoted as ${\mathcal {D}}^{{\mathbf {E}}}$ and is called **embedded** in ${\mathcal {D}}$ (Statnikov et al. 2013). The target or outcome variable will be referred to as *T*. For the reader’s convenience, we summarize all acronyms and mathematical notation used throughout the paper in Table 1.

**Table 1:** Common acronyms and mathematical notation with a short description

FBS	Forward–Backward Selection algorithm
TFBS	Template for Forward–Backward Selection
TIE*	Target Information Equivalence algorithm
TMFBS	Template for Multiple Forward–Backward Selection
PEQ	Performance Equivalence of solutions
MEQ	Model Equivalence of solutions
IEQ	Information Equivalence of solutions
MSG	Multiple Solutions Graph
*X*	random variable
${\mathbf {Z}}$	set of random variables
${\mathbf {Z}}_{1:n}$	subset of ${\mathbf {Z}}$ with the first *n* variables
${\mathcal {D}}$	dataset - 2-D matrix
*T*	outcome (or target) variable
${\mathbf {F}}$	variables in ${\mathcal {D}}$ except *T*
${\mathcal {D}}^{\mathbf {E}}$	embedded dataset without variables ${\mathbf {E}}$
${\mathbf {X}} {\perp } {\mathbf {Y}} \ | \ {\mathbf {Z}}$	${\mathbf {X}}$ and ${\mathbf {Y}}$ are conditionally independent given ${\mathbf {Z}}$
$\textsc {Test}({\mathbf {X}};{\mathbf {Y}}|{\mathbf {Z}})$	conditional independence test of ${\mathbf {X}}$ with ${\mathbf {Y}}$ given ${\mathbf {Z}}$
$\textsc {Pvalue}({\mathbf {X}};{\mathbf {Y}}|{\mathbf {Z}})$	p-value $\textsc {Test}({\mathbf {X}};{\mathbf {Y}}|{\mathbf {Z}})$
$\alpha$	significance level threshold
${\mathbf {S}}$	reference solution (set of features) to the single feature selection problem
${\mathbf {M}}$	set of multiple solutions equivalent to ${\mathbf {S}}$
${\mathcal {H}}$	predictive algorithm (or learner)
${\mathcal {H}}^*(T|{\mathbf {X}})$	theoretically optimal model ${\mathcal {H}}$ for *T* using features ${\mathbf {X}}$
$\hat{{\mathcal {H}}}(T|{\mathbf {X}})$	model trained by ${\mathcal {H}}$ on dataset ${\mathcal {D}}$
$E[{\mathcal {L}}({\mathcal {H}}^*(T|{\mathbf {X}}))]$	expected loss of ${\mathcal {H}}^*(T|{\mathbf {X}})$ w.r.t. the true conditional distribution of *T* given ${\mathbf {X}}$
${\mathbf {C}}$	set of candidates returned by OrderVariables
${\mathcal {G}}$	a directed acyclic graph
*s*, *t*	root and leaf of ${\mathcal {G}}$
*p*	a directed path from *s* to *t* in ${\mathcal {G}}$
$N_i$	*i*-th node of ${\mathcal {G}}$
$var[N_i]$	set of variables associated with $N_i$
$parents[N_i]$	set of parents of $N_i$
$children[N_i]$	set of children of $N_i$

### Conditional independence

Conditional independence of variables ${\mathbf {X}}$ and ${\mathbf {Y}}$ given ${\mathbf {Z}}$ is denoted as ${\mathbf {X}} {\perp } {\mathbf {Y}} \ | \ {\mathbf {Z}}$, the test of conditional independence is denoted as $\textsc {Test}({\mathbf {X}}; {\mathbf {Y}} | {\mathbf {Z}})$, while the *p*-value returned by the test is denoted as $\textsc {Pvalue}({\mathbf {X}}; {\mathbf {Y}} | {\mathbf {Z}})$. Two variables are deemed conditionally dependent (independent) if the *p*-value of the test is below (above) a pre-specified significance level $\alpha$.

Tests of conditional independence can be used to perform feature selection; see Definition 1 of Markov blankets for the relation of conditional independence and the optimal solution to the feature selection problem. Examples of such methods are the Grow-Shrink feature selection algorithm (Margaritis and Thrun 2000; Margaritis 2009), the Max-Min Parents and Children algorithm (Tsamardinos et al. 2003a), HITON (Aliferis et al. 2003) and the recently introduced Forward–Backward selection with Early Dropping algorithm (Borboudakis and Tsamardinos 2019).

A general method for conditional independence testing is using nested likelihood-ratio tests (Wilks 1938) (or asymptotically equivalent approximations thereof such as F tests or score tests). For a given statistical model (e.g., logistic or linear regression), a likelihood-ratio test for conditional independence of *X* and ${\mathbf {Y}}$ given ${\mathbf {Z}}$ can be performed by fitting two models for *X*, one using ${\mathbf {Z}}$ and one using ${\mathbf {Y}} \cup {\mathbf {Z}}$. The test statistic is computed as the difference in deviances, and is distributed as a $\chi ^2$ with degrees of freedom equal to the difference in number of parameters between the models (Wilks 1938). For example, the G-test for conditional independence (Agresti 2002) is a likelihood-ratio test for multinomial data, while the partial correlation test (Fisher 1924) is a test for multivariate Gaussian data, which is asymptotically equivalent to a likelihood-ratio test using linear regression (Christensen 2011). Interested readers may refer to Sections 2.2 and 6.2 in Tsamardinos et al. (2019) for a more detailed description of likelihood-ratio tests and how to implement them.

We note that the nested likelihood-ratio method assumes that the larger hypothesis (statistical model) is correctly specified, i.e., that it can correctly model the data distribution. If the model is misspecified, the statistic follows a different distribution (Foutz and Srivastava 1977). Methods to deal with model misspecification have been proposed in (White 1982; Vuong 1989). Hereafter, we will assume that the models are correctly specified, and that the conditional independence tests can correctly identify dependencies and independencies. In practice this assumption is often violated, especially for continuous data, where testing conditional independence is particularly hard (Shah and Peters 2018) (in fact, it is shown that there is no uniformly valid test). However, it is very useful for theoretical analysis, and is common for proving correctness of algorithms using conditional independence tests (see Theorem 4 b in (Aliferis et al. 2010) for example).

### The single and multiple feature selection problems

The **solution** ${\mathbf {S}}$ to the single feature selection problem can be defined as *identifying a minimal-size subset of the variables that is optimally predictive for an outcome variable* *T* *of interest* (Tsamardinos and Aliferis 2003), and is also called the **Markov blanket** of *T* (Koller and Sahami 1996).

#### Definition 1

(*Markov Blanket*) A Markov blanket ${\mathbf {S}}$ of *T* is defined as ${\mathbf {S}} = \mathop {{{\,\mathrm{argmin}\,}}}\limits _{|\mathbf {S'}|} \{\mathbf {S'} \subseteq {\mathbf {F}} : T {\perp } {\mathbf {F}} \setminus \mathbf {S'} \ | \ \mathbf {S'}\}$, that is, ${\mathbf {S}}$ is a minimal-size subset of ${\mathbf {F}}$ that renders *T* conditionally independent of all variables not in ${\mathbf {S}}$.

The above definitions only consider a single solution to the feature selection problem. A more general definition, accounting for the possibility of multiple solutions follows.

#### Definition 2

(*Multiple Feature Selection Problem*) Let ${\mathbf {S}}$ be the solution to the single feature selection problem (called the **reference** solution). The solution ${\mathbf {M}}$ to the multiple feature selection problem consists of all minimal-size sets $\mathbf {S_i} \subseteq {\mathbf {F}}$ that are statistically equivalent to ${\mathbf {S}}$.

There are several ways to define and test statistical equivalence between feature sets, each of which can lead to a different solution set ${\mathbf {M}}$ to the above problem, which will be addressed in Sect. 4.

### The JKP taxonomy of features

John et al. (1994) classify features into three categories: **strongly relevant** (also called indispensable), **weakly relevant** and **irrelevant** features; we will refer to this as the JKP taxonomy hereafter, based on the initials of the authors names (John, Kohavi and Pfleger).

#### Definition 3

(*Strongly Relevant Feature*) A feature *X* is strongly relevant for *T* if $T {\not \perp } X \ | \ {\mathbf {F}} \setminus \{X\}$.

#### Definition 4

(*Weakly Relevant Feature*) A feature *X* is weakly relevant for *T* if $T {\perp } X \ | \ {\mathbf {F}} \setminus \{X\} \wedge \exists {\mathbf {Z}} \subseteq {\mathbf {F}} \setminus \{X\}, T {\not \perp } X \ | \ {\mathbf {Z}}$.

#### Definition 5

(*Irrelevant Feature*) A feature *X* is irrelevant for *T* if $\forall {\mathbf {Z}} \subseteq {\mathbf {F}} \setminus \{X\}, T {\perp } X \ | \ {\mathbf {Z}}$.

Intuitively, a feature *X* is strongly relevant if it still provides additional predictive information for *T* given (conditioned on) all other features, weakly relevant if it is not strongly relevant but still provides information for *T* that is redundant given other features (*X* is informative for *T* given some subset ${\mathbf {Z}}$), and irrelevant if it does not provide any information for *T* (*X* is uninformative for *T* given any subset ${\mathbf {Z}}$).

One would expect that an optimal feature selection method would only return the strongly relevant features and filter out both the weakly relevant and the irrelevant features, as they do not provide additional information in the presence of all strongly relevant features. This is also what is suggested by the terminology of “strongly” and “weakly”. In other words, one would expect that the strongly relevant features to correspond to the Markov Blanket features. Indeed, this is correct but only when there is a single, unique solution. However, as we describe in the next section, when the solution to the problem is not unique, then there is not a single Markov Blanket and correspondence of strongly relevant features with the Markov Blanket breaks down.

## A taxonomy of features in the presence of multiple solutions

In the presence of multiple solutions, the JKP taxonomy of features (John et al. 1994) is counter-intuitive and misleading. Intuitively, we’d expect that keeping only the strongly relevant features should be enough for optimal prediction using an optimal classifier, as weakly relevant features are superfluous. Consider however the case where, feature *X* is in a reference solution and $X'$ is a copy of *X* (or a one-to-one deterministic transformation). Now, both features are weakly relevant (one makes the other redundant); one expects that both should be filtered out (not selected). However, at least one of them should be selected for optimal prediction. In fact, if all members of a reference solution have a copy in the dataset, then no feature is strongly relevant for the given problem, which is counter-intuitive. The problem stems from the fact that the JKP taxonomy does not distinguish between weakly relevant features that carry superfluous information, and features that carry information necessary for optimal prediction but this information component is shared among many features or feature subsets. The above considerations led us to define the following taxonomy of features:

### Definition 6

(*Irrelevant Feature*) A feature *X* is irrelevant if it provides no information for *T* in any context ${\mathbf {Z}}$, i.e., if $\forall {\mathbf {Z}} \subseteq {\mathbf {F}} \setminus \{X\}, T {\perp } X \ | \ {\mathbf {Z}}$.

### Definition 7

(*Indispensable Feature*) A feature *X* is indispensable if it belongs in all solutions for *T*, i.e., $\forall {\mathbf {S}}_i \in {\mathbf {M}}, X \in {\mathbf {S}}_i$.

### Definition 8

(*Replaceable Feature*) A feature *X* is replaceable if it is not indispensable and belongs in some solution for *T*, i.e., $\exists {\mathbf {S}}_i, {\mathbf {S}}_j \in {\mathbf {M}}, {\mathbf {S}}_i \ne {\mathbf {S}}_j \wedge X \in {\mathbf {S}}_i \wedge X \not \in {\mathbf {S}}_j$.

### Definition 9

(*Redundant Feature*) A feature *X* is redundant if it provides information for *T* in some context but does not belong in any solution, i.e., $\exists {\mathbf {Z}} \subseteq {\mathbf {F}} \setminus \{X\}, T {\not \perp } X \ | \ {\mathbf {Z}} \wedge \forall {\mathbf {S}}_i \in {\mathbf {M}}, X \not \in {\mathbf {S}}_i$.

The proposed taxonomy is related to the JKP taxonomy as follows: (a) irrelevant features are defined the same in both taxonomies, (b) strongly relevant features coincide with indispensable features, (c) a weakly relevant feature is called replaceable if it is present in some solution, and is called redundant otherwise. Thus, when there is a single solution, then irrelevant, strongly relevant, and weakly relevant features coincide with irrelevant, indispensable, and redundant features respectively.

## Statistically equivalent feature sets

In this section, we will provide several definitions and tests for statistical equivalence of feature sets. Such tests are used by the main algorithm proposed in Sect. 6 for identifying multiple statistically equivalent solutions. Specifically, given the reference solution ${\mathbf {S}}$ (see Definition 2 of the multiple feature selection problem), tests of feature set equivalence can be used to identify the set of solutions ${\mathbf {M}}$ which are statistically equivalent to ${\mathbf {S}}$. We review methods from related statistical literature on model selection, and show how they can be used to test different types of statistical equivalence of feature sets. Finally, we provide some advice for performing such tests in practice.

We proceed with some definitions used hereafter. Let ${\mathcal {H}}$ be a predictive algorithm (also called learner). Examples are linear and logistic regression, as well as support vector machines and random forests, where hyper-parameter values (such as the kernel and cost for support vector machines) are fixed. For a given target *T* and set of random features ${\mathbf {X}}$, we denote with ${\mathcal {H}}^*(T|{\mathbf {X}})$ the optimal model attainable in the sample limit using algorithm ${\mathcal {H}}$ and features ${\mathbf {X}}$ for predicting *T*. Given a dataset ${\mathcal {D}}$ sampled from the joint distribution of *T* and ${\mathbf {F}}$ (the set of all features), we denote with $\hat{{\mathcal {H}}}(T|{\mathbf {X}})$ the model obtained by training ${\mathcal {H}}$ on the dataset ${\mathcal {D}}$ for *T* using only features ${\mathbf {X}}$. Let ${\mathcal {L}}$ be a loss (or performance) function, such as the squared error for regression tasks and the deviance for probabilistic classification (such as logistic regression), or penalized versions of the above such as the Akaike (Akaike 1973) and Bayesian information criteria (Schwarz et al. 1978). We use $E[{\mathcal {L}}({\mathcal {H}}^*(T|{\mathbf {X}}))]$ to denote the expected loss of ${\mathcal {H}}^*(T|{\mathbf {X}})$ with respect to the true conditional distribution of *T* given ${\mathbf {X}}$.

### Definitions of statistical equivalence of feature sets

We proceed by listing different definitions for assessing equivalence of feature sets. Tests for them are presented in the subsequent section.

#### Definition 10

(*Performance Equivalence* (**PEQ**) (*see also Fig. 11 in* (Statnikov et al. 2013)) Feature sets ${\mathbf {X}}$ and ${\mathbf {Y}}$ are performance equivalent relative to an algorithm ${\mathcal {H}}$ and loss function ${\mathcal {L}}$ if $E[{\mathcal {L}}({\mathcal {H}}^*(T|{\mathbf {X}}))] = E[{\mathcal {L}}({\mathcal {H}}^*(T|{\mathbf {Y}}))]$.

PEQ requires that the optimal predictive models obtained by two sets features have the same expected loss, and depends both on ${\mathcal {H}}$ and ${\mathcal {L}}$. A drawback of performance equivalent feature sets is that it is not guaranteed that they are Markov blankets (i.e., optimal solutions). PEQ can hold even if the feature sets predict separate parts of the outcome’s distribution equally well. For example, let $X \backsim {\mathcal {N}}(0,1)$, $Y \backsim {\mathcal {N}}(0,1)$ and $T = X + Y$. It can be seen that the feature sets $\{X\}$ and $\{Y\}$ are PEQ for *T* relative to any performance measure and linear models. However, none of them is a Markov blanket; the Markov blanket of *T* is the union of both feature sets. Another problematic case is if the loss function used is not a proper scoring function, i.e., if the minimum loss is not achieved for optimal feature sets. This can be the case for performance functions such as the classification accuracy. For example, let *T* and *X* be binary features, and let $P(T = 1|X = 1) = 0.9$, $P(T = 1| X = 0) = 0.6$ and $P(X = 1) = 0.5$. Any optimal model optimizing accuracy would always predict $T = 1$, irrespective of the value of *X*, attaining an accuracy of $75\%$. Thus, both feature sets $\{\emptyset \}$ and $\{X\}$ are performance equivalent using classification accuracy. However, only $\{X\}$ is a Markov blanket, as *X* clearly gives information about *T* (*X* is dependent with *T*). Therefore, if the main goal is knowledge discovery, the PEQ definition for identifying equivalences may not be preferable.

#### Definition 11

(*Model Equivalence* (**MEQ**)) Feature sets ${\mathbf {X}}$ and ${\mathbf {Y}}$ are model equivalent relative to algorithm ${\mathcal {H}}$ if ${\mathcal {H}}^*(T|{\mathbf {X}})$ and ${\mathcal {H}}^*(T|{\mathbf {Y}})$ make the same predictions for any sample of the j.p.d. of ${\mathbf {X}} \cup {\mathbf {Y}}$.

MEQ requires that *T* can be modeled using ${\mathcal {H}}$ equally well with either set of features. Note that, given the same algorithm ${\mathcal {H}}$, MEQ implies PEQ, regardless of the loss function ${\mathcal {L}}$. Naturally, the reverse does not necessarily hold.

#### Definition 12

(*Information Equivalence* (**IEQ**))^1^ Feature sets ${\mathbf {X}}$ and ${\mathbf {Y}}$ are information equivalent if both $T {\perp } {\mathbf {X}} \ | \ {\mathbf {Y}}$ and $T {\perp } {\mathbf {Y}} \ | \ {\mathbf {X}}$ hold.

IEQ (Lemeire 2007; Statnikov et al. 2013) is the strictest definition of equivalence, and is independent of both ${\mathcal {H}}$ and ${\mathcal {L}}$. It requires that feature sets are **interchangeable**: they have to contain the same information about the outcome. For example, let $X \backsim {\mathcal {N}}(0,1)$, $Y = \beta X$ and $T = X + Y$. In this case we say that $\{X\}$ and $\{Y\}$ are information equivalent, as *Y* depends deterministically on *X*. Intuitively, choosing any of them gives the same information about *T*. In the taxonomy proposed previously, *X* and *Y* are called replaceable.

While in theory IEQ does not depend on ${\mathcal {H}}$, in practice the definition of IEQ may also implicitly depend on some predictive algorithm. The reason is that the tests of conditional independence used for testing IEQ also use a predictive algorithm or statistical model internally (e.g., the logistic regression algorithm used by nested likelihood-ratio tests). Because of that, it is not clear if and under which conditions MEQ and IEQ differ, or whether IEQ always implies MEQ, assuming both use the same algorithm ${\mathcal {H}}$; if not, it is trivial to construct examples were only one of MEQ and IEQ holds.

Surprisingly, *even for the same algorithm* ${\mathcal {H}}$, *MEQ does not always imply IEQ*. This happens if combining both feature sets leads to a better set of features (i.e., if the feature sets were not Markov blankets). For example, let *X*, *Y* and *W* be binary variables, and $T = W \vee (X \oplus Y)$, where $\oplus$ is the logical XOR operator. Assuming an optimal classifier ${\mathcal {H}}$, $\{X,W\}$ and $\{Y,W\}$ are MEQ relative to ${\mathcal {H}}$, as knowing only *X* or *Y* does not provide information for *T*, and thus models constructed using either variable set will give the same predictions based on *W*. The feature sets are not IEQ, as *X* and *Y* provide information for *T* conditional on *X* or *Y* respectively. The reason for the above is that neither of the feature sets is a Markov blanket, and combining them leads to a better feature set (which is the Markov blanket in this example).

### Testing statistical equivalence of feature sets and its relation to the model selection problem

The problem of model selection can be defined as identifying the best fitting model out of a set of candidate models. Most approaches address the more general problem of selecting among two or more competing models (e.g., choosing between a normal and log-normal distribution), whereas in our case we are interested in the special case of comparing two sets of features relative to the same model (e.g., selecting the best feature set for linear regression); we will focus on the latter hereafter. Testing for equivalence is related to model selection, as it deals with the problem of identifying models that fit the data equally well instead of identifying the better one. We will briefly mention some approaches that are related to the problems of equivalence testing; interested readers may refer to (Pesaran and Weeks 1999) for an in-depth overview, as well as a discussion on the similarities of model selection and hypothesis testing.

#### Vuong’s variance test

In the context of model selection, Vuong (1989) proposed the **variance test** to test if two models fit the data equally well, that is, if they are MEQ. The statistic is computed as the variance of the log likelihood-ratio^2^ of the models (that is, the variance of the difference of log likelihoods). Let $\mathcal {LL}$ denote the log-likelihood function. For two sets of features ${\mathbf {X}}$ and ${\mathbf {Y}}$, and predictive algorithm ${\mathcal {H}}$, the statistic is defined as follows.

$$\begin{aligned} \text {Statistic} \equiv \text {var}[\mathcal {LL}({\mathcal {H}}^*(T|{\mathbf {X}})) - \mathcal {LL}({\mathcal {H}}^*(T|{\mathbf {Y}}))] \end{aligned}$$

The variance is defined with respect to the true joint distribution of *T*, ${\mathbf {X}}$ and ${\mathbf {Y}}$. In practice it can be estimated using the sample variance of the log-likelihood ratio of the estimated models $\hat{{\mathcal {H}}}(T|{\mathbf {X}})$ and $\hat{{\mathcal {H}}}(T|{\mathbf {Y}})$. In case MEQ holds the statistic equals zero, and follows a sum of scaled chi-squares otherwise. As no closed-form expression for a sum of scaled chi-squares exist, it is hard to compute. Furthermore, in practice the statistic is also hard to estimate accurately, especially for small sample sizes (Shi 2015). One way to overcome those problems is to use resampling-based methods (see Section 7.2 in Pesaran and Weeks (1999) for an example of using bootstrap tests (Efron and Tibshirani 1994)). A disadvantage of resampling-based methods such as bootstrapping is that it requires fitting two models and computing their log-likelihood for each bootstrap sample, which is very computationally demanding. Another, computationally faster approach, is to use a permutation test instead; the test is described in more detail in Sect. 4.3.

#### The comprehensive approach

Atkinson (1970) proposed the **comprehensive** approach, which constructs a third model that contains both initial models as special cases. When comparing feature sets relative to the same model, this reduces to creating a third model $\hat{{\mathcal {H}}}(T|{\mathbf {X}} \cup {\mathbf {Y}})$, and testing whether it provides additional information compared to $\hat{{\mathcal {H}}}(T|{\mathbf {X}})$ and $\hat{{\mathcal {H}}}(T|{\mathbf {Y}})$ (see Section 3.1 in (Atkinson 1970)). This is done by performing two likelihood-ratio tests, comparing the original models with the combined model. In case the tests are not rejected, the sets ${\mathbf {X}}$ and ${\mathbf {Y}}$ can be considered equivalent. Note that, the likelihood-ratio tests between $\hat{{\mathcal {H}}}(T|{\mathbf {X}} \cup {\mathbf {Y}})$ and the models $\hat{{\mathcal {H}}}(T|{\mathbf {X}})$ and $\hat{{\mathcal {H}}}(T|{\mathbf {Y}})$ corresponds to the conditional independence tests $\textsc {Test}(T; {\mathbf {Y}} | {\mathbf {X}})$ and $\textsc {Test}(T; {\mathbf {X}} | {\mathbf {Y}})$ respectively (assuming the models are correctly specified; see Sect. 2.1). Thus, this is a direct application of the IEQ definition using likelihood-ratio based conditional independence tests.

#### The J-test

Davidson and MacKinnon (Davidson and MacKinnon 1981; MacKinnon 1983) propose several methods for choosing among two competing models, similar in spirit to Atkinson’s approach. One such method is the J-test, which we describe next. Let $\hat{{\mathcal {P}}}(T|{\mathbf {X}})$ denote the predictions of model ${\hat{H}}(T|{\mathbf {X}})$ on the dataset ${\mathcal {D}}$ used for learning it. The idea of the J-test is similar to the comprehensive approach, but instead of testing $\textsc {Test}(T; {\mathbf {X}} | {\mathbf {Y}})$ and $\textsc {Test}(T; {\mathbf {Y}} | {\mathbf {X}})$, the J-test tests $\textsc {Test}(T; \hat{{\mathcal {P}}}(T|{\mathbf {X}})| {\mathbf {Y}})$ and $\textsc {Test}(T; \hat{{\mathcal {P}}}(T|{\mathbf {Y}}) | {\mathbf {X}})$, (i.e., it tests whether the predictions of the models provide additional information for *T*). Basically, the J-test uses the predictions of the model as proxies for the information provided by the feature sets. That way, it avoids fitting models using the union of feature sets, and may have higher power than the comprehensive approach. However, it may not be as accurate, as it only uses the predictions of one set instead of all features, and thus interactions between feature sets ${\mathbf {X}}$ and ${\mathbf {Y}}$ may be missed. Thus, it can be seen as a more sample efficient alternative to the comprehensive approach for (approximately) testing IEQ. An overview of the J-test can be found in (Bremmer 2003).

#### Paired two-sample tests

Other methods for testing PEQ or MEQ, not based on model selection, are paired two-sample tests such as paired t-tests or the Wilcoxon signed rank test. MEQ can be tested by comparing the predictions of models ${\hat{H}}(T|{\mathbf {X}})$ and ${\hat{H}}(T|{\mathbf {Y}})$, while PEQ can be tested using the losses of each prediction. We note that, the latter is only applicable for loss functions which can be computed on a per-sample basis (e.g., mean squared error), and thus is not applicable for measures like the area under the ROC curve.

### Practical considerations and recommendations

#### Discovering multiple Markov blankets

Although all definitions and tests of statistical equivalence are for arbitrary feature sets and not restricted to Markov blankets, in practice we are often interested in identifying multiple solutions that are Markov blankets, i.e., solving the multiple feature selection problem (see Definition 2). Recall that the solution to the multiple feature selection problem ${\mathbf {M}}$ consists of all feature sets that are statistically equivalent to the reference solution ${\mathbf {S}}$ (a solution to the single feature selection problem).

In order to identify multiple Markov blankets, it is necessary to be able to test whether a feature set ${\mathbf {X}}$ is a Markov blanket. We will assume that ${\mathbf {X}}$ already is minimal, i.e., no features can be removed from it without losing information about *T*. Given a reference solution ${\mathbf {S}}$ (which is a Markov blanket by definition), a way to test whether ${\mathbf {X}}$ is a Markov blanket is by performing a test of statistical equivalence with ${\mathbf {S}}$. Depending on the type of equivalence (PEQ, MEQ and IEQ) there are different guarantees for ${\mathbf {X}}$. If ${\mathbf {X}}$ is a Markov blanket all equivalences are implied, but the opposite is only guaranteed for IEQ. Thus, *when the goal is identification of multiple Markov blankets, we recommend using tests of information equivalence*.

#### Tests with feature sets that are not Markov blankets

While theoretically the reference solution should be a Markov blanket, in practice this might not be the case due to statistical errors or violations of assumptions of the tests. A reference solution that is not a Markov blanket may lead to missing candidate solutions which are Markov blankets, as they provide more information about the target than the reference solution. We note that the algorithm will still return solutions that are equivalent to the reference solution.

Another problem is if the reference is a Markov blanket, and a candidate solution ${\mathbf {X}}$ is a superset of a Markov blanket (i.e., not minimal). Technically ${\mathbf {X}}$ contains the same information as any Markov blanket, and therefore might be identified as equivalent to the reference solution. This might be acceptable for some practical applications (e.g., if the focus is predictive performance), but not for applications requiring interpretability.

Both of the above are hard to detect in practice. *We propose to perform extensive hyper-parameter tuning*, in order to identify reasonably good approximations of Markov blankets, minimizing the chance of the above problems occurring.

#### Power of IEQ tests

Recall that in order to perform a test of IEQ between two feature sets ${\mathbf {X}}$ and ${\mathbf {Y}}$, one has to perform two tests on all of the features ($\textsc {Test}(T; {\mathbf {Y}} | {\mathbf {X}})$ and $\textsc {Test}(T; {\mathbf {X}} | {\mathbf {Y}})$). For instance, when likelihood-ratio tests are used, a model using the union of features ${\mathbf {X}} \cup {\mathbf {Y}}$ has to be created to test for IEQ. Because of that, tests of IEQ require significantly larger sample sizes than PEQ or MEQ tests, which do not fit models using the union of feature sets. When tests of IEQ are performed with small sample sizes, they may not have enough power to distinguish between non-equivalent feature sets, and will falsely consider them equivalent. To avoid this problem, *we recommend using tests for PEQ or MEQ as a “safe lock” before applying the IEQ test*. To further reduce false positives, we recommend to combine the above with a high significance level (relative to the available sample size) for all tests.

#### Reliability of PEQ and MEQ tests

To improve the reliability of the two-sample and variance tests (due to violations of assumptions or low sample size) we recommend using permutation-based variants instead. Another advantage is that permutation tests are not limited to any specific class of loss functions, and can be also applied to performance measures such as the area under the ROC curve, which are computed using the a vector of predictions. Under the null hypothesis of PEQ or MEQ, the losses or predictions can be permuted across paired samples (i.e., exchange randomly whether a prediction comes from model ${\hat{H}}(T|{\mathbf {X}})$ or ${\hat{H}}(T|{\mathbf {Y}}$)). Thus, a permutation test can be performed as follows: (a) compute the statistic *s* on the original sample, (b) randomly permute the input vectors (predictions or losses) across the same (paired) samples, each one with probability 0.5, (c) compute the statistic $s_i$ (of the *i*-th permutation) of interest on the permuted sample, and (d) repeat (b-c) *B* times (e.g., 1000 times). For the variance test, the *p*-value is then computed as the proportion of permutation statistics (including the statistic on the original sample from step (a) (Davison and Hinkley 1997)) smaller than or equal to the sample statistic *s*

$$\begin{aligned} p\text {-value} \equiv \frac{1 + \sum _{i = 1}^{B} I(s_i \le s)}{B + 1} \end{aligned}$$

where *I* is the indicator function.

#### Summary

For knowledge discovery purposes, we recommend aiming for information equivalent solutions. In order to reduce the chance of false positive equivalences, we recommend to (a) perform extensive tuning of the hyper-parameters^3^ of the feature selection algorithm, in order to increase the chance of identifying Markov blankets, (b) first apply a permutation-based variance test for PEQ or MEQ to quickly filter out false equivalences and (c) afterwards apply an IEQ test using the comprehensive approach to decide for equivalence, and (d) use relatively high significance levels to further reduce the number of false positives. In anecdotal experiments we found that all of the above were important to reduce the number of non-equivalent solutions, which in many cases was extremely high otherwise.

## A general template for forward–backward algorithms


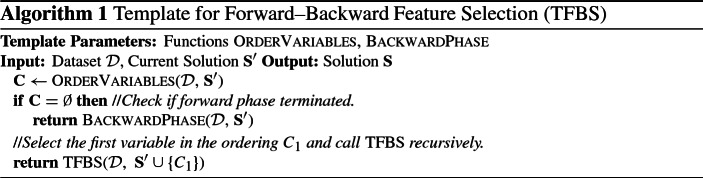
 
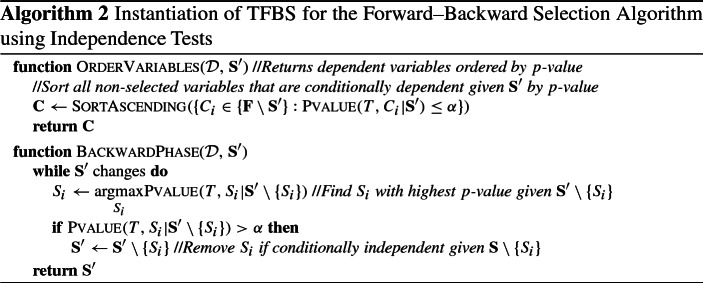


We propose a general template for greedy feature selection algorithms, called **Template for Forward–Backward Feature Selection** (TFBS), which we will later extend to select multiple, statistically equivalent solutions. This template can express a class of stepwise methods, namely algorithms that consist of two phases: (a) a greedy forward phase, where features are selected one at a time, and (b) an optional backward phase, applied after the forward phase terminates, to remove false positives.

The algorithm is shown in Algorithm 1, and will be referred to as TFBS hereafter. We present a recursive version of the algorithm, as it will lead to a natural extension for multiple solutions. For the sake of brevity, constant input arguments like the target variable *T* and hyper-parameter values are omitted. TFBS has two main components: (a) a variable ordering strategy OrderVariables, and (b) a function BackwardPhase that performs the backward phase of the algorithm. In order for those functions to be **admissible**, they have to satisfy the following conditions. OrderVariables must return an ordered set of candidate variables ${\mathbf {C}}$ for selection, such that: (a) ${\mathbf {C}}$ is empty if no more variables should be selected, (i.e., if $\mathbf {S'} \supseteq {\mathbf {S}}$) (b) ${\mathbf {C}}$ does not contain any already selected variable (i.e., ${\mathbf {C}} \cap \mathbf {S'} = \emptyset$), and (c) ${\mathbf {C}}$ contains all variables that could be selected at that iteration, in order of preference. An alternative way to look at (c) is that any variable $C_{j+1}$ would be selected if the algorithm were to be executed on the embedded dataset ${\mathcal {D}}^{C_{1:j}}$ (i.e., after excluding variables $C_{1:j}$). The BackwardPhase function must remove all and only the false positive variables. We note that, for the single solution case OrderVariables does not have to provide a complete ordering, but can only return the next variable to select. That presentation was chosen to allow for an easier extension for multiple solutions.

A large class of feature selection algorithms can be expressed as instantiations of TFBS. Examples include the forward–backward selection (FBS) algorithm (Kutner et al. 2004; Weisberg 2005) and variations or extensions of it (Margaritis and Thrun 2000; Tsamardinos et al. 2003b; Margaritis 2009), information-theoretic feature selection methods (Brown et al. 2012), as well as causal-based algorithms (Aliferis et al. 2010) like the MMPC (Tsamardinos et al. 2003a) and HITON-PC (Aliferis et al. 2003) algorithms. An instantiation of FBS using *p*-values of conditional independence tests to order variables is shown in Algorithm 2. The forward phase tests for each variable $C_i \in \{{\mathbf {F}} \setminus \mathbf {S'}\}$ if it is dependent with *T* given $\mathbf {S'}$, and selects the one with the lowest *p*-value. Thus, the OrderVariables function for FBS returns all variables that are conditionally dependent dependent given the current set of selected variables $\mathbf {S'}$ in ascending order of *p*-values. The backward phase removes at each iteration the least dependent variable given all selected variables (i.e., the one with the highest *p*-value which is higher than the significance level $\alpha$), until no more variables can be removed.

## Extending TFBS for multiple solutions

The forward phase of TFBS can be seen as a search on the space of feature sets (Kohavi and John 1997). Each state of the search space contains a set of selected variables $\mathbf {S'}$, and its neighbors are all states which additionally contain one of the variables in ${\mathbf {C}}$. As the search is only in one direction (i.e., only when variables are added), we will refer to the neighbors of a state *t* containing an extra variable as its **children**, the previous state as its **parent**, and all children of its parent (except for *t* itself) as its **siblings**. Thus, TFBS traverses that search space by only visiting the first child (i.e., the one where $C_1$ is selected). Given this view, we use a simple idea to extend it for multiple solutions: instead of exploring a single child at each iteration, we use **backtracking** (Russell and Norvig 2003) to explore all children and consider multiple solutions. A candidate solution is then returned if it is equivalent with the reference solution (i.e., the one obtained by TFBS).

**Fig. 1:**
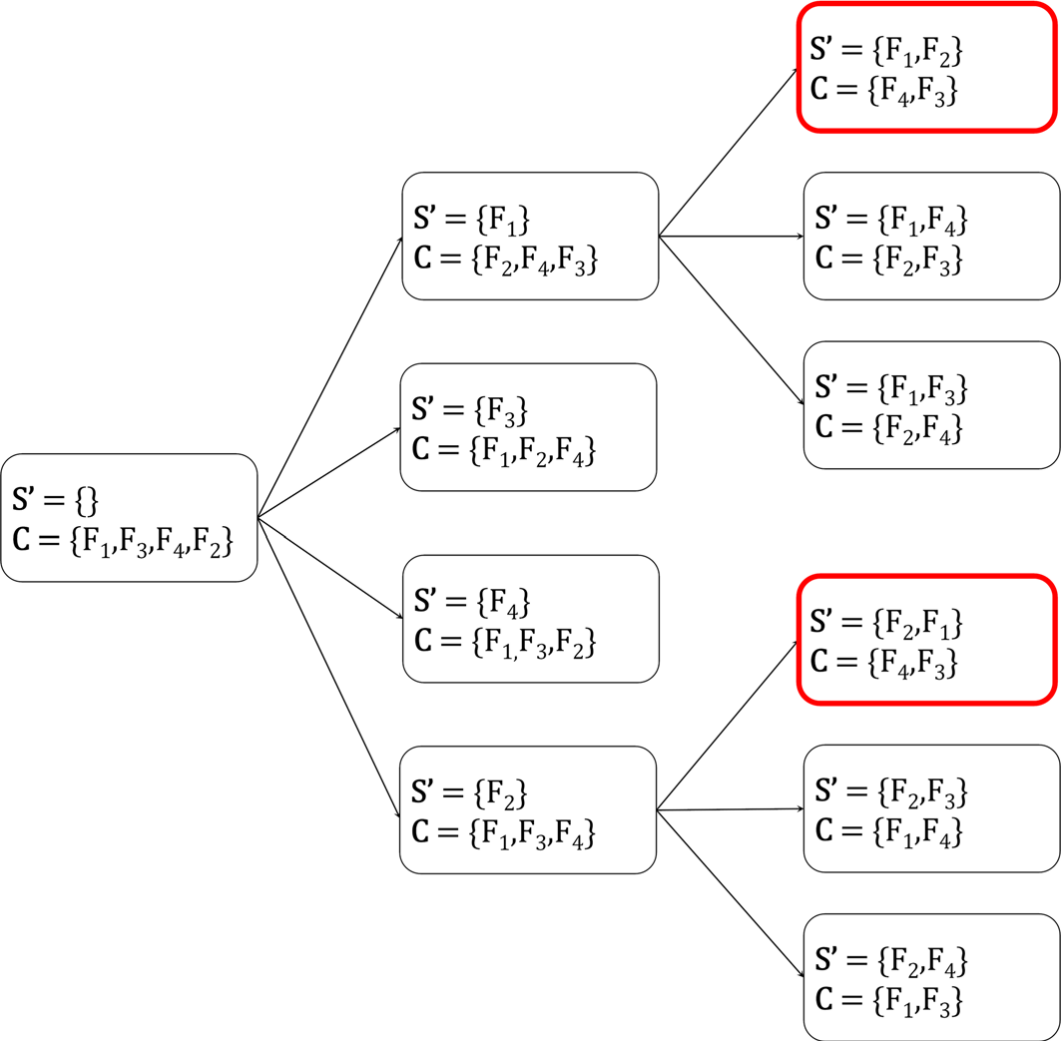
An example showing that naive backtracking can explore the same state twice. The set of currently selected variables is denoted as $\mathbf {S'}$, and ${\mathbf {C}}$ denotes the set of candidate variables returned by OrderVariables using $\mathbf {S'}$. For simplicity, we consider only 4 variables, assume that OrderVariables does not remove any variables, and only show part of the search space. We can see that there are two states (highlighted in red) with the exact same set of selected variables (Color figure online)

The naive approach is not very practical, as it may consider the same solutions multiple times. For example, the state containing $\mathbf {S'} = \{X,Y\}$ can be reached twice by selecting the variables in different order. In general, each solution can be obtained in *m*! ways, where *m* is the size of the solution. Thus, in the worst case, up to *p*! solutions (where *p* is the number of variables) may be considered, even though there are only $2^p$ unique combinations! An example is shown in Fig. 1. Next, we propose a strategy to avoid such repetition.

### A strategy to avoid repeating states

**Fig. 2:**
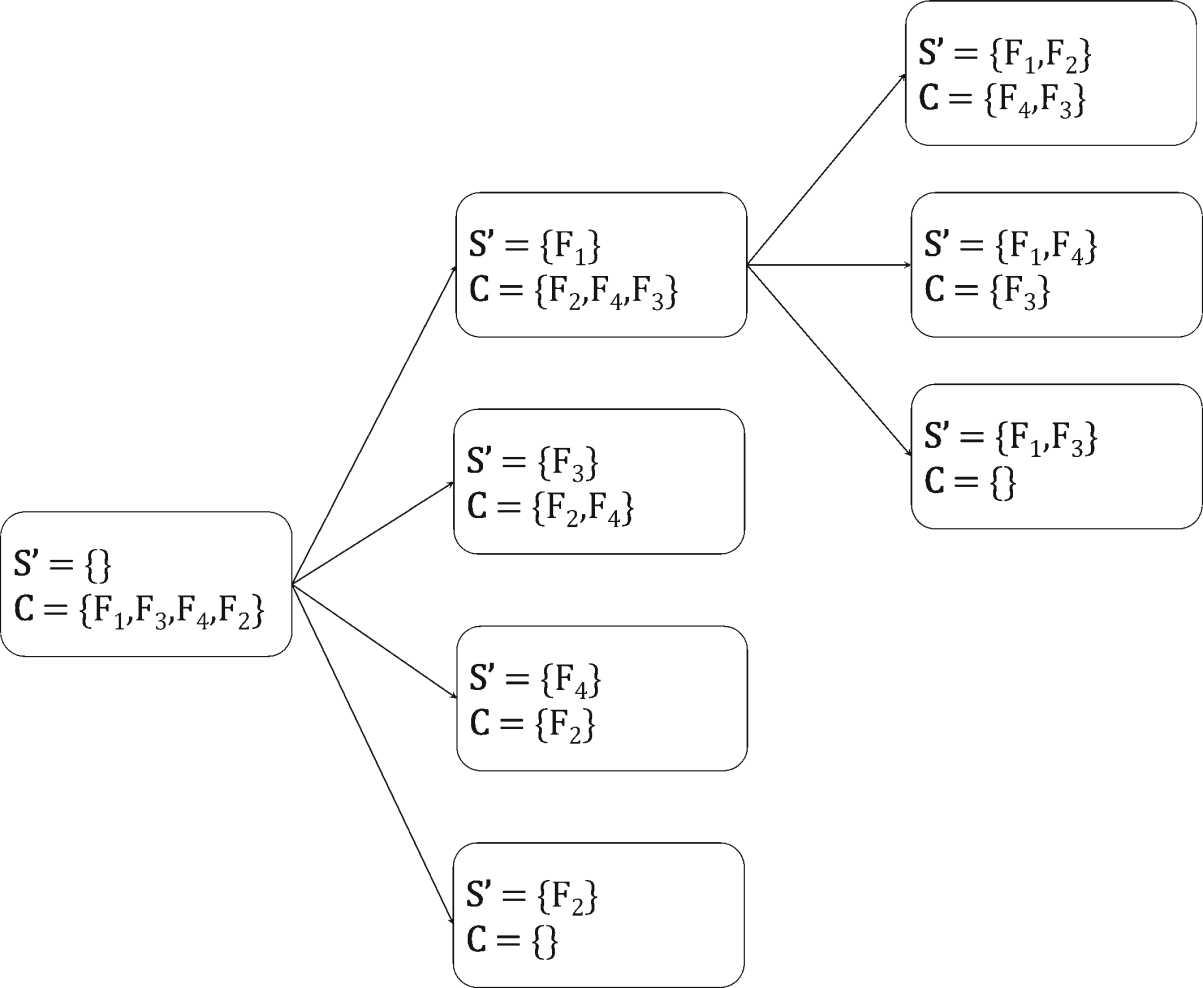
An example showing how the proposed strategy can avoid repeating states on the example considered in Fig. 1. Note that the set of candidate variables ${\mathbf {C}}$ of any state does not contain the selected variables of any of its siblings that come before that (i.e., are above it). For example, variable $F_1$ is selected only once (top state in the middle column), and variable sets containing it are explored only in its children states, but not on any of its siblings. However, variable $F_2$ which is selected in the top right state is also considered for selection in the bottom state, as they are neither children nor siblings of each other

Let $\mathbf {S'}$ be the current set of selected variables, ${\mathbf {C}} = \{C_1, \ldots , C_k\}$ be the set of candidate variables returned by OrderVariables and $\{t_1, \ldots , t_k\}$ be the respective states obtained after selecting one of the variables in ${\mathbf {C}}$. To avoid repeating states, each variable $C_i$ is **excluded** from consideration in all subsequent sibling states of $t_i$ (i.e., $t_{i+1}, \ldots , t_k$). Therefore, $t_{i+1}, \ldots , t_k$ will never lead to the same feature sets as $t_i$, as $C_i$ is selected in all explored child states of $t_i$ but in none of the ones explored by $t_{i+1}, \ldots , t_k$. On a more intuitive level, once $C_i$ is selected and all children states of $t_i$ are fully explored, the algorithm is given the opportunity to consider all feature sets that contain $\mathbf {S'} \cup \{C_i\}$, and thus there is no need to further consider $C_i$ from that point on. Note that, $C_i$ may still be considered in other tree branches which contain a different set of variables, i.e., where the set of variables is not a subset of $\mathbf {S'}$. Figure 2 shows how this strategy can avoid repeating states in the previous example of Fig. 1.

The above strategy is equivalent to executing the algorithm twice with different input datasets, starting both times with the set of selected variables initialized to $\mathbf {S'}$: once with ${\mathcal {D}}$ (which contains $C_i$), and once with the embedded dataset ${\mathcal {D}}^{C_i}$ (which does not contain $C_i$). This can be shown by simply noting that, up to that point, the algorithm would select the exact same variables $\mathbf {S'}$ using ${\mathcal {D}}^{C_i}$, and then would select $C_{i+1}$ instead of $C_i$, as $C_i$ is not contained in the dataset (unless of course $C_i$ was the last variable, in which case it would terminate). The above observation is summarized in the below.

#### Lemma 1

Let ${\mathcal {D}}$ be the input dataset, $\mathbf {S'}$ be the current set of selected variables, ${\mathbf {C}} = \{C_1, \ldots , C_k\}$ be the current set of candidate variables and $\{t_1, \ldots , t_k\}$ be the respective states obtained after selecting one of the variables in ${\mathbf {C}}$. Excluding $C_i$ from consideration from states $t_{i+1}, \ldots , t_k$ is equivalent to re-running the algorithm on the embedded dataset ${\mathcal {D}}^{C_i}$.

### The TMFBS algorithm for multiple solutions


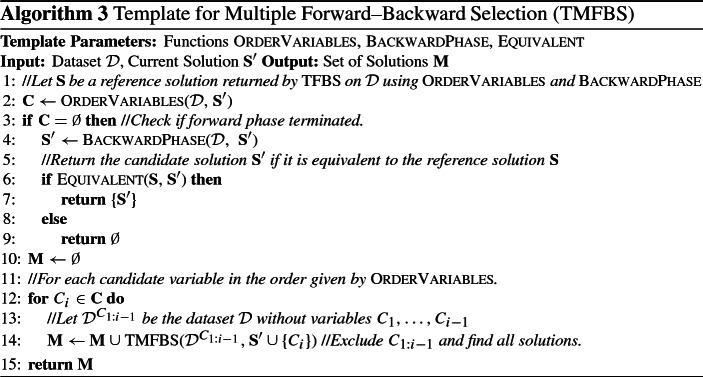


We propose the **Template for Multiple Forward–Backward Selection** algorithm (TMFBS), an extension of TFBS which uses backtracking, as well as the proposed strategy for avoiding repeated states, in order to identify multiple statistically equivalent solutions. For each identified candidate solution, TMFBS performs a test for statistical equivalence of feature sets, to test if it is equivalent with the reference solution ${\mathbf {S}}$, which is assumed to be known. In practice, ${\mathbf {S}}$ is initialized to the first solution identified during the search (which coincides with the reference solution returned by TFBS). The algorithm is shown in Algorithm 3.

#### Theoretical properties of TMFBS

We proceed with the theoretical properties of TMFBS. Let ${\mathcal {A}}$ be an admissible pair of functions $\langle \textsc {OrderVariables}, \textsc {BackwardPhase} \rangle$. We show that TMFBS identifies all equivalent solutions if the following assumption^4^ holds.

##### Assumption 1

TFBS instantiated with ${\mathcal {A}}$ identifies the Markov blanket of *T* in any dataset ${\mathcal {D}}$.

It is important to notice that Assumption 1 refers to *any* dataset ${\mathcal {D}}$, and therefore implies that TFBS using ${\mathcal {A}}$ can also find a Markov blanket of *T* in any embedded dataset in ${\mathcal {D}}$ (or any dataset which contains ${\mathcal {D}}$); however, note that a Markov blanket in an embedded dataset is not necessarily a Markov blanket in the original ${\mathcal {D}}$.

Depending on ${\mathcal {A}}$, the distributions for which Assumption 1 holds differ. For example, for FBS^5^ it has been shown that Assumption 1 holds (assuming an oracle for testing conditional independence) for distributions that satisfy the local composition property (Statnikov et al. 2013), i.e., if $T {\perp } {\mathbf {X}} \ | \ {\mathbf {Z}} \wedge T {\perp } {\mathbf {Y}} \ | \ {\mathbf {Z}} \Rightarrow T {\perp } {\mathbf {X}} \cup {\mathbf {Y}} \ | \ {\mathbf {Z}}$ holds for any sets ${\mathbf {X}}, {\mathbf {Y}}$ and ${\mathbf {Z}}$. Conditions under which HITON-PC (Aliferis et al. 2003) identifies all solutions are given in (Statnikov et al. 2013). We note that, there is no general recipe to identify for which distributions Assumption 1 holds for arbitrary ${\mathcal {A}}$. However, for algorithms that can be connected to probabilistic graphical models (such as FBS and HITON-PC), one can use the theory of probabilistic graphical models as a guide to find conditions under which it holds (see (Statnikov et al. 2013)). We proceed with the main result.

##### Theorem 1

TMFBS using ${\mathcal {A}}$ will identify all and only the solutions equivalent with the reference solution in any dataset ${\mathcal {D}}$, if (a) ${\mathcal {A}}$ satisfies Assumption 1 and, (b) it has access to an oracle for deciding equivalence.

##### Proof

Let ${\mathbf {S}} \subseteq {\mathbf {F}}$ be an arbitrary solution equivalent to the reference solution. We will show inductively that any such solution can be obtained by running TMFBS.

Let ${\mathbf {S}}^j$ be the current solution after *j* steps, and let ${\mathbf {C}}^j$ be the corresponding candidate variables returned by $\textsc {OrderVariables}$ given ${\mathbf {S}}^j$. Assume that ${\mathbf {S}}^j \subseteq {\mathbf {S}}$. We will show that, if progress can be made (i.e., if ${\mathbf {S}}^j$ is not a solution), then there is a neighbor state such that ${\mathbf {S}}^{j+1} \subseteq {\mathbf {S}}$.

If ${\mathbf {S}}^j$ equals ${\mathbf {S}}$, then ${\mathbf {C}}^j$ is empty (condition (a) of admissibility of), and the algorithm would terminate and return the solution. We will prove the ${\mathbf {S}}^j \subset {\mathbf {S}}$ case by contradiction. Assume that ${\mathbf {C}}^j$ does not contain any variable of ${\mathbf {S}}$. Because of that, ${\mathbf {C}}^j$ could be excluded without altering the solution, which is equivalent to executing the algorithm on ${\mathcal {D}}^{{\mathbf {C}}^j}$ by Lemma 9. After *j* steps this would lead to a state where ${\mathbf {C}}^j$ is empty, and the algorithm would terminate. However, as ${\mathbf {S}}^j$ is a subset of ${\mathbf {S}}$ it can’t be a solution, given that ${\mathbf {S}}$ is a solution, otherwise ${\mathbf {S}}$ wouldn’t be minimal. This would violate Assumption 10 for ${\mathcal {D}}^{{\mathbf {C}}^j}$ which contains ${\mathbf {S}}$, leading to a contradiction. Therefore ${\mathbf {C}}^j$ must contain some variable from ${\mathbf {S}} \setminus {\mathbf {S}}^j$, and consequently there is a neighbor state such that, after $j+1$ steps, ${\mathbf {S}}^{j+1} \subseteq {\mathbf {S}}$ holds.

Finally, because TMFBS has access to an oracle for deciding statistical equivalence, any false positive solutions identified during the search will be discarded, retaining only equivalent solutions. $\square$

#### Sound rules for pruning the search space

In this section we will introduce several sound rules for speeding TMFBS up, which are direct consequences of Theorem 1, and will prove their correctness.

Theorem 1 states that TMFBS will find all solutions in any ${\mathcal {D}}$, and therefore also in any embedded dataset ${\mathcal {D}}^{{\mathbf {E}}}$ in ${\mathcal {D}}$. An immediate consequence of this is that, if no solution is found in some dataset ${\mathcal {D}}^{{\mathbf {E}}}$, no solution can be found in any embedded dataset of ${\mathcal {D}}^{{\mathbf {E}} \cup \mathbf {E'}}$; if there was one in ${\mathcal {D}}^{{\mathbf {E}} \cup \mathbf {E'}}$, it would also be contained ${\mathcal {D}}^{{\mathbf {E}}}$ as ${\mathcal {D}}^{{\mathbf {E}} \cup \mathbf {E'}}$ is embedded in ${\mathcal {D}}^{{\mathbf {E}}}$.

##### Corollary 1

Let ${\mathcal {D}}^{{\mathbf {E}}}$ and ${\mathcal {D}}^{{\mathbf {E}} \cup \mathbf {E'}}$ be two datasets, where the latter is embedded in the former. If no equivalent solution is contained in ${\mathcal {D}}^{{\mathbf {E}}}$, then no equivalent solution is contained in ${\mathcal {D}}^{{\mathbf {E}} \cup \mathbf {E'}}$.

Based on this, we propose and incorporate the following rules in TMFBS for pruning the search space and speeding it up.

##### Pruning Rule 1

If TMFBS(${\mathcal {D}}^{C_{1:i-1}}$, ${\mathbf {S}} \cup \{C_{i}\}$) does not return any equivalent solution, stop and return ${\mathbf {M}}$.

##### Pruning Rule 2

Before calling TMFBS(${\mathcal {D}}^{C_{1:i-1}}$, ${\mathbf {S}} \cup \{C_{i}\}$), check if for some ${\mathcal {D}}^{{\mathbf {E}}}$, ${\mathbf {E}} \subseteq C_{1:i-1}$ no equivalent solution was returned, and if so stop and return ${\mathbf {M}}$.

In TMFBS, Rule 2 is checked before the recursive call to TMFBS(${\mathcal {D}}^{C_{1:i-1}}$, ${\mathbf {S}} \cup \{C_{i}\}$) (Line 14 in Algorithm 3), while Rule 1 is checked afterwards. We note that Rule 2 is one of the conditions of the IGS procedure used by TIE* (see fourth bullet and step 1 of Figure 9 in (Statnikov et al. 2013)).

Recall that, after including a variable $C_i$ in state $t_i$, none of its subsequent siblings $t_{i+1}, \ldots , t_k$ will consider $C_i$ again, and thus increasingly smaller embedded datasets are explored. Thus, if TMFBS(${\mathcal {D}}^{C_{1:i-1}}$, ${\mathbf {S}} \cup \{C_{i}\}$) does not lead to a solution, by Corollary 1 neither can any call to TMFBS with datasets embedded in ${\mathcal {D}}^{C_{1:i-1}}$. Although Rule 1 identifies many cases implied by Corollary 1 that can be pruned, it does not necessarily identify all of them. Combining it with Rule 2, which is basically a direct application of Corollary 1, ensures completeness. We note that, theoretically Rule 1 is not required; however, in contrast to Rule 2, which requires to keep track of all embedded datasets that did not lead to any solution, Rule 1 can be implemented trivially and efficiently.

The previous rules only consider the forward phase of TMFBS. We identified another pruning rule, which regards the backward phase of the algorithm.

##### Pruning Rule 3

If no solution returned by TMFBS(${\mathcal {D}}^{C_{1:i-1}}$, ${\mathbf {S}} \cup \{C_{i}\}$) contains $C_i$, stop and return ${\mathbf {M}}$.

Rule 3 is checked after the recursive call to TMFBS(${\mathcal {D}}^{C_{1:i-1}}$, ${\mathbf {S}} \cup \{C_{i}\}$) (after Line 14 in Algorithm 3).

Pruning Rule 3 regards cases where variable $C_i$ is selected at some step but is not in any solution in ${\mathcal {D}}^{C_{1:i-1}}$, which can happen if $C_i$ is removed during the backward phase from all of them. Basically, if including $C_i$ leads to some solutions (i.e., Rule 1 does not apply), but none of them actually contains $C_i$, $C_i$ was a false positive which got removed by the backward phase. Thus, the call TMFBS(${\mathcal {D}}^{C_{1:i-1}}$, ${\mathbf {S}} \cup \{C_{i}\}$) would give the same results as TMFBS(${\mathcal {D}}^{C_{1:i}}$, ${\mathbf {S}}$), which is the same as the results of all remaining states in the current recursive call (i.e., TMFBS(${\mathcal {D}}^{C_{1:i}}$, ${\mathbf {S}} \cup \{C_{i+1}\}$), $\ldots$, TMFBS(${\mathcal {D}}^{C_{1:k-1}}$, ${\mathbf {S}} \cup \{C_{k}\}$)) Because of that, there is no reason to consider the remaining candidates $C_{i+1:k}$, which have already been implicitly considered, and ${\mathbf {M}}$ can be returned.

Despite all attempts to speed-up TMFBS, the number of candidate solutions may still be exponential in the number of variables, and thus TMFBS may not terminate in a reasonable time frame. *This is not a weakness of TMFBS, but an inherent property of the problem*. Thus, to avoid such cases in practice, we recommend setting a limit on the number of candidate states to consider, the number of solutions to return, or a combination of both. This problem also motivated us to develop an algorithm for summarizing and visualizing multiple solutions, presented in Sect. 7.

#### Computational complexity

In this section we present an analysis of the complexity of TMFBS. Due to the nature and inherent intractability of the problem, it is hard to quantify accurately and in a useful manner the computational complexity of TMFBS. The complexity depends on many factors, such as (a) the cost of OrderVariables and BackwardPhase for the specific instantiation of TMFBS, (b) the cost of testing whether a candidate solution is equivalent to the reference solution using Equivalent, (c) the search strategy of TMFBS induced by OrderVariables, which affects when TMFBS encounters equivalent solutions, (d) the total number of solutions, (e) the size of solutions, (f) whether the pruning rules trigger or not, and (g) the properties of the input dataset (e.g., number of samples and variables). To simplify analysis, we will make the following assumptions.

First, the costs of BackwardPhase and Equivalent are negligible compared to the cost of the forward phase, which is dominated by OrderVariables. We use *f*(*n*) to denote the time taken by OrderVariables to rank *n* variables. To make things even simpler, we will implicitly assume that *n* is set to the total number of variables in the input dataset, and will simply refer to the cost as *f*. Also, in practice the backward phase typically removes only a small number of variables, compared to the ones selected during the forward phase (see also Section 4.4 in (Statnikov et al. 2013)). Given the above, and because pruning rule 3 will trigger for variables which were removed during the backward phase, avoiding further search, *we will only focus on variables which were added during the forward phase and are not removed afterwards*. Next, *we will ignore any pruning*, unless stated otherwise, as this depends on OrderVariables and the search space it produces. Finally, we will use an upper bound on the size of the candidate solutions, denoted as *L*, allowing us to focus on the worst case scenario. We proceed with the analysis.

**Finding the next candidate solution**. An interesting and useful way to look at the problem, is to measure the time required for identifying a new candidate solution given the previous candidate solution at a search tree node *N*. The exact cost depends on the location of *N* on the search tree. First, note that after backtracking the algorithm will continue from some node on the path from the root of the search tree to *N*. Each such node already contains a set of selected variables, with the size depending on the position of the node on the path (e.g., the *i*-th node already has *i* selected variables). As the maximum size of any candidate solution is *L*, the cost of finding the next candidate solution is between *f* and $L \cdot f$. Specifically, for the *i*-th node on the path, the cost is $(L-i) \cdot f$.

**Proving there are no more solutions**. Another interesting aspect is the cost of deciding whether there are additional equivalent solutions. Again, assume that TMFBS arrived at some root node *N*. TMFBS will backtrack from the leaf node *N* to the root node, one node at a time. In case there are no more solutions, TMFBS will explore at most one candidate solution from each node to some leaf node, as the pruning rules will trigger. As each node on the from the root to *N* is considered, the total cost equals $\sum _{i=1}^{L} i \cdot f = O(L^2 \cdot f)$. Using this result, one can show that, in case of a single solution, the total cost equals the cost of finding the reference solution ($O(L \cdot f)$) plus the cost of proving there are no more solutions, i.e., $O(L^2 \cdot f)$.

### Relation to the TIE* algorithm


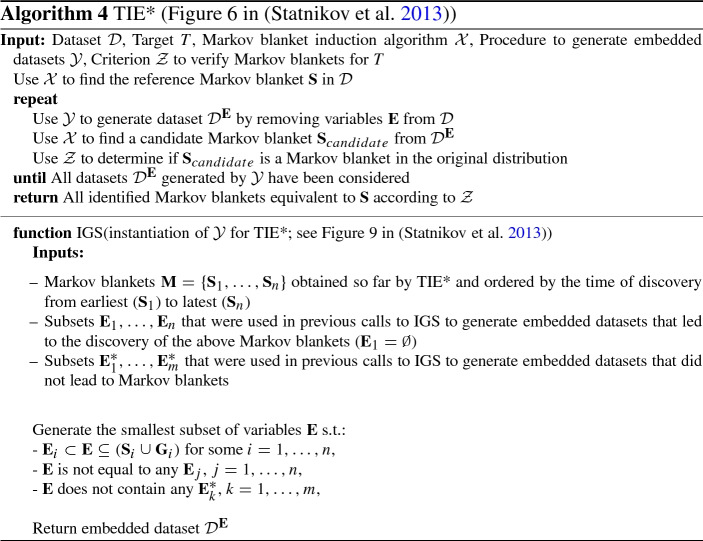


TIE* (Statnikov et al. 2013) is another general algorithm for identifying multiple solutions. It uses three components: (a) a Markov blanket discovery algorithm ${\mathcal {X}}$, (b) a function ${\mathcal {Y}}$ that generates embedded datasets, and (c) a criterion ${\mathcal {Z}}$ that tests if a solution is a Markov blanket in the original distribution. The TIE* algorithm, along with the IGS method for creating embedded datasets (adapted to use similar notation with TMFBS) are shown in Algorithm 4. As a criterion ${\mathcal {Z}}$, any test for equivalence of feature sets can be used.

The main idea of TIE* is to (a) identify the reference solution ${\mathbf {S}}$ on the original dataset ${\mathcal {D}}$, (b) remove variables of ${\mathbf {S}}$ from ${\mathcal {D}}$ and run ${\mathcal {X}}$ on the embedded datasets to identify additional solutions, and (c) repeat (a,b) for each new solution found, until no more solutions can be found. All of this is done without running ${\mathcal {X}}$ on a dataset (a) more than once, and (b) if no solution was found in a superset of the dataset (same as Pruning Rule 2). We proceed with a comparison to TMFBS.

TIE* and TMFBS have the same theoretical properties, that is, both identify all equivalent solutions if Assumption 1 holds and they have access to an oracle for deciding equivalence.^6^ The main advantage of TIE* is that it is more general than TMFBS, in the sense that it can be used with any Markov blanket discovery algorithm that satisfies Assumption 1, while TMFBS is limited to forward–backward type methods. We’d like to remind the reader that many popular algorithms can be expressed with TFBS (see Sect. 5 for examples), making this limitation less restrictive in practice. On the other hand, because TMFBS is a more specialized algorithm than TIE*, it can take advantage of the specific search strategy of the underlying Markov blanket algorithm used. This allows TMFBS to safely avoid re-computation of conditional independence tests, and to forget any unneeded results, using the fact that the search strategy is a greedy forward–backward type.

TIE* could also avoid re-computing conditional independence tests between different runs of the underlying Markov blanket algorithm. However, in order to be able to handle any such algorithm, using any arbitrary order of performing the tests, the only way to avoid re-computation is by caching the results of the tests. This is also proposed by the authors in Section 4.4 in (Statnikov et al. 2013). Unfortunately, the number of tests could increase polynomially with the number of features leading to large computational overheads in storing and retrieving from the cache, as well as large memory overheads.

Finally, we note that implementing TIE* efficiently is not trivial. For example, the choice of data structures and algorithms to implement algorithm ${\mathcal {Y}}$ for generating embedded datasets in TIE* is particularly important for the algorithm to become practical.

Next, we take a closer look at the computational costs of TMFBS and TIE*. In order to compare the algorithms, we assume that ${\mathcal {X}}$ used by TIE* fits into the TFBS framework, i.e., it uses $\textsc {OrderVariables}$ internally to select the next variable. For simplicity, we will also assume that both algorithms explore all candidate solutions in the same order, and that no pruning is performed. We use *f* to denote the cost of $\textsc {OrderVariables}$, ignoring the number of input variables. Recall that TMFBS requires between 1 and *L* calls to $\textsc {OrderVariables}$ for identifying a new candidate solution. TIE* on the other hand needs to execute ${\mathcal {X}}$ from scratch, and therefore always requires *L* calls. If there is only a single solution, both algorithms have a cost of $O(L^2 \cdot f)$. We proceed with a worst case scenario, assuming a balanced search tree with a maximum depth of *L* and that the whole search tree is explored. Note that TMFBS will call $\textsc {OrderVariables}$ exactly once for each node on the search tree. The number of nodes in a balanced tree of depth *L* can be computed as the partial sum of the geometric sequence $\sum _{l=0}^L n^L = \frac{n^{L+1} - 1}{n - 1} = O(n^L)$, where *n* is the total number of variables. Therefore, the cost of TMFBS is $O(f \cdot n^L)$. This is interesting as, even though the cost of finding a new candidate solution is between *O*(*f*) and $O(L \cdot f)$ for TMFBS, on average it is *O*(*f*) (one call to OrderVariables for each candidate). The complexity of TIE* is $O(L \cdot f \cdot n^L)$, as there are $n^L$ leaf nodes (i.e., the number of candidate solutions) in a balanced tree of depth, and each call of ${\mathbf {X}}$ has a cost of $O(L \cdot f)$. While it is unlikely that the search tree will have that form, the example demonstrates that the speed-up of TMFBS over TIE* is not trivial. We’d like to point out that the analysis favors TIE* over TMFBS, as we assumed that TIE* explores the same candidate solutions as TMFBS and in the same order. The search strategy of TMFBS is dynamic and depends how variables are ranked by $\textsc {OrderVariables}$ at each step, the input dataset and the set of already selected variables. In contrast, the algorithms for generating embedded datasets for TIE* (i.e., its search strategy) lead to predetermined search strategies. We argue that this gives TMFBS an edge over TIE*, as any reasonable $\textsc {OrderVariables}$ function is likely to lead to a more efficient exploration of the search space in practice.

Finally, we would like to note that TMFBS is not a simple instantiation of TIE*. While TMFBS can be technically expressed using TIE*, by running TMFBS and using the results ${\mathbf {M}}$ in ${\mathcal {Y}}$ to generate embedded datasets (one dataset per Markov blanket, containing only those variables), there is no natural way achieve that. TMFBS could perhaps be derived from TIE* by having ${\mathcal {X}}$ and ${\mathcal {Y}}$ communicate their internal state in order to simulate TMFBS, but this would be non-trivial and would require the introduction of additional constructs and data structures (e.g., cache).

## Summarizing and visualizing multiple solutions

The more solutions are identified, the harder it is to interpret them. Typically, most solutions have some overlap (features that are indispensable) and only differ for a few features (replaceable features), enabling a more compact representation. We propose a data structure for compactly representing feature sets, as well as an algorithm to construct it next.

### Multiple solution graphs

**Fig. 3:**
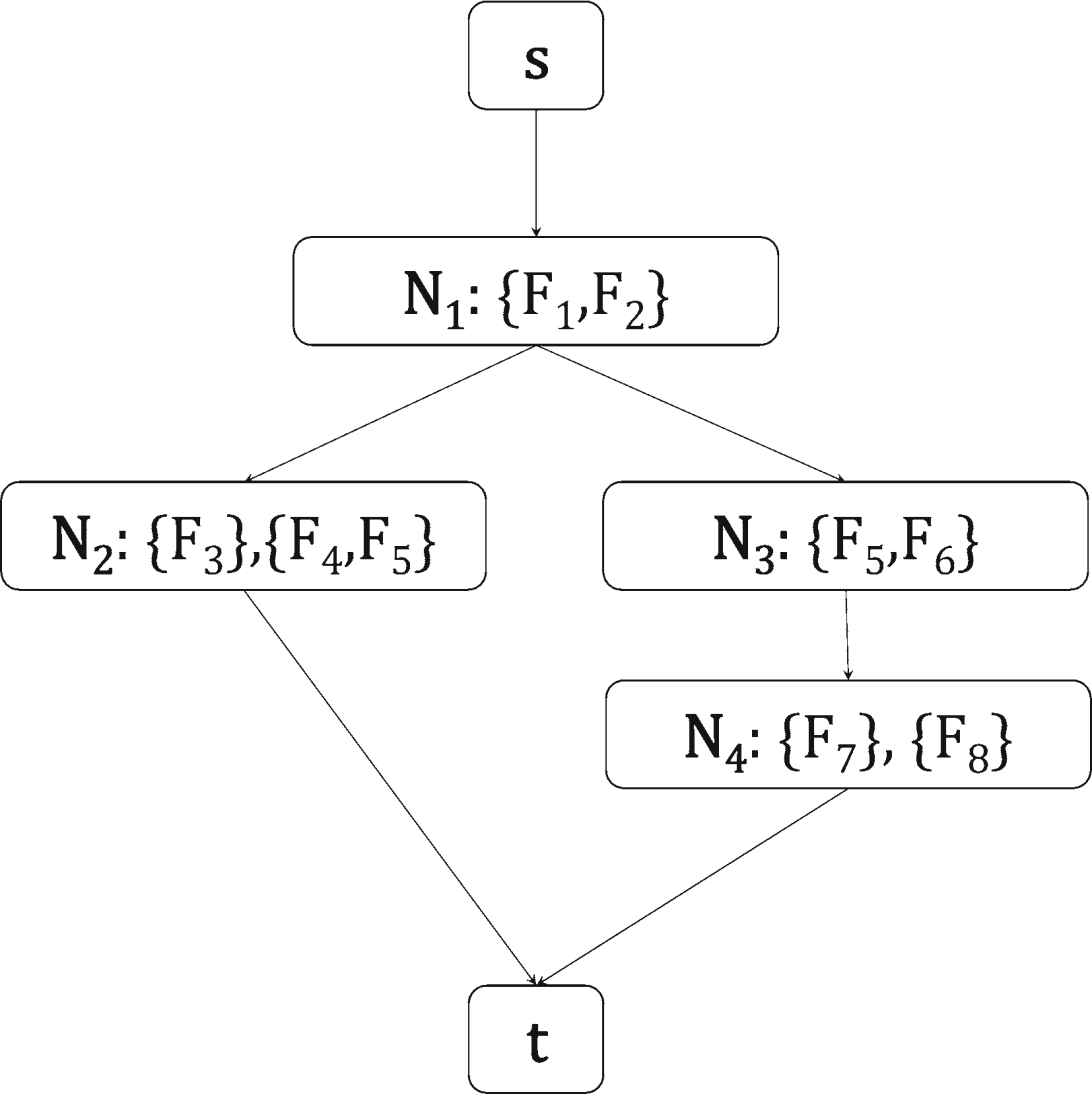
An example of a multiple solution graph. All nodes except *s* and *t* contain one or multiple feature sets. As nodes with multiple features sets represent OR relations, solutions can be reconstructed by taking a path from *s* to *t* and picking exactly one feature set from each node. This MSG has two paths from *s* to *t*, and encodes 4 solutions in total: $\{F_1, F_2, F_3\}$, $\{F_1, F_2, F_4, F_5\}$, $\{F_1, F_2, F_5, F_6, F_7\}$, and $\{F_1, F_2, F_5, F_6, F_8\}$

Let ${\mathbf {M}}$ denote a set of solutions ${\mathbf {M}} = \{\mathbf {S_1}, \ldots , \mathbf {S_k}\}$. We propose to represent them with a **multiple solution graph** (MSG), which is a directed acyclic graph ${\mathcal {G}}$ with the following properties: (a) ${\mathcal {G}}$ contains exactly one root and leaf node, called *s* and *t*, (b) each other node in ${\mathcal {G}}$ is associated with one or more sets of features, and has in and out degree at least one (c) each directed path *p* from *s* to *t* represents a solution, which can be obtained by choosing one of the feature sets of each node on *p* and taking the union of the chosen feature sets, and (d) ${\mathcal {G}}$ does not encode any additional solutions. An example of an MSG is shown in Fig. 3.

Hereafter, we will use the names $N_i$ to refer to the *i*-th node in ${\mathcal {G}}$ and $var[N_i]$ to refer to the sets of features associated with $N_i$. In case $var[N_i]$ contains only a single set of features, it will directly refer to that set. If it is clear from the context, we will refer to a node with its associated feature set. We will use $parents[N_i]$ and $children[N_i]$ to refer to the parents and children of $N_i$ in ${\mathcal {G}}$ respectively.

### An algorithm for constructing multiple solutions graphs


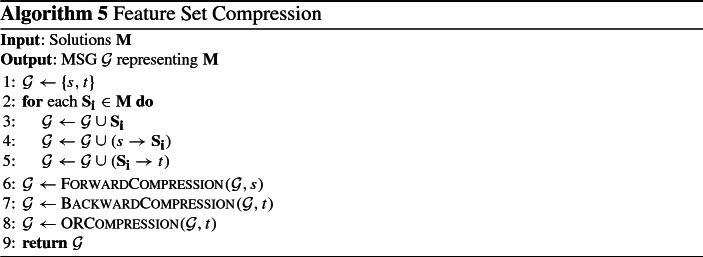


We propose a greedy algorithm to construct an MSG ${\mathcal {G}}$ to compactly represent a set of solutions ${\mathbf {M}} = \{\mathbf {S_1} \ldots , \mathbf {S_k}\}$ (see Algorithm 5). First, *k* nodes are created, one for each feature set in ${\mathbf {M}}$. Then, edges from *s* into each of those nodes, as well as edges into *t* out of them are included in ${\mathcal {G}}$. It is easy to see that ${\mathcal {G}}$ exactly represents ${\mathbf {M}}$, as it contains *k* paths from *s* to *t*, one for each feature set. Afterwards, forward and backward compression steps are performed to reduce the size of ${\mathcal {G}}$. Both are performing operations on ${\mathcal {G}}$ with the goal of simplifying it. Until that step, all nodes contain a single feature set. The final step is to merge nodes, creating nodes that contain multiple feature sets. Before describing everything in detail, we proceed by presenting the operations used, along with proofs of correctness.

### Compression operations

#### Operation 1

(Forward Merging) Let ${\mathbf {N}} = N_1, \ldots , N_n$ be a set of nodes. If $\mathbf {F'} = \bigcap _{N_i} var[N_i] \ne \emptyset$ and all of them have exactly the same set of parents ${\mathbf {P}}$, then, a new node $N'$ is created with $var[N'] =\mathbf {F'}$, $parents[N'] = {\mathbf {P}}$, $children[N'] = {\mathbf {N}}$, and remove all incoming edges from ${\mathbf {N}}$, as well as all features $\mathbf {F'}$ from ${\mathbf {N}}$.

#### Operation 2

(Backward Merging) Let ${\mathbf {N}} = N_1, \ldots , N_n$ be a set of nodes. If $\mathbf {F'} = \bigcap _{N_i} var[N_i] \ne \emptyset$ and all of them have exactly the same set of children ${\mathbf {C}}$, then, a new node $N'$ is created with $var[N'] = \mathbf {F'}$, $parents[N'] = {\mathbf {N}}$, $children[N'] = {\mathbf {C}}$, and remove all outgoing edges from ${\mathbf {N}}$, as well as all features $\mathbf {F'}$ from ${\mathbf {N}}$.

#### Operation 3

(OR Merging) Let ${\mathbf {N}} = N_1, \ldots , N_n$ be a set of nodes. If all nodes have the same sets of parents ${\mathbf {P}}$ and children ${\mathbf {C}}$, then, a new node $N'$ is created with $var[N'] = \{var[N_1]$, $\ldots$, $var[N_n]\}$, add edges from ${\mathbf {P}}$ to $N'$ as well as edges from $N'$ to ${\mathbf {C}}$, and remove all nodes ${\mathbf {N}}$ from ${\mathcal {G}}$.

**Fig. 4:**
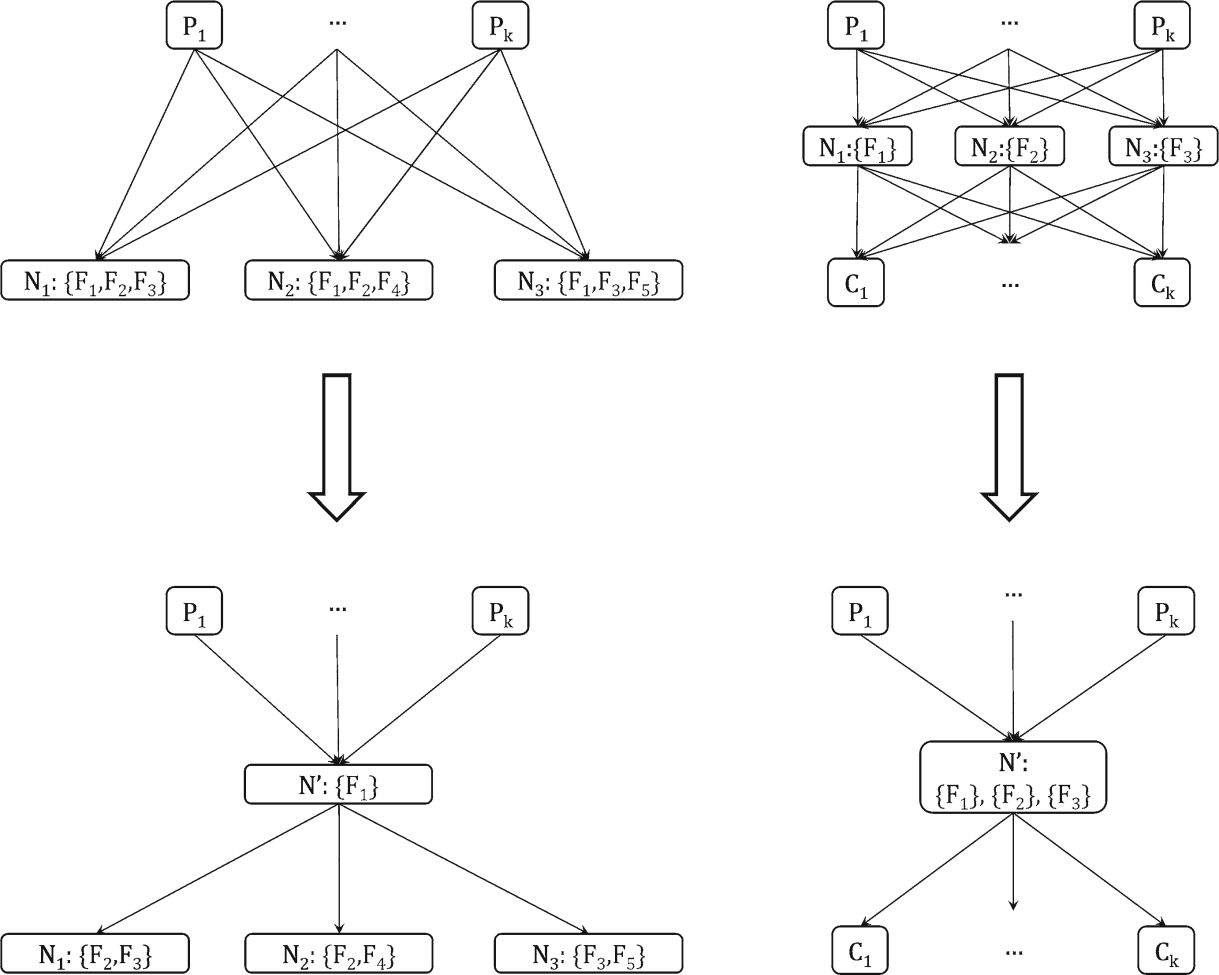
Examples of the forward merging (left) and OR merging (right) operations

Figure 4 shows examples of the forward merging and OR merging operations; the backward merging operation is not shown, as it is symmetric to the forward merging operation, requiring common children instead of common parents. In the example of the forward merging operation, the nodes $N_1$, $N_2$ and $N_3$ share the same parents and have the feature $F_1$ in common. The resulting node $N'$ contains $F_1$, which is removed from the feature sets of the other nodes. The OR merging operation requires that the nodes share the same parents and children. If so, the nodes can be merged into a single node, containing as feature sets all features sets of the merged nodes, as shown in the example. As can be seen in the previous examples, all operations simplify the graph by removing nodes, features and edges. Next we will quantify those effects for all operations.

We start with the forward and backward merging operations. Without loss of generality, we consider the forward merging operation; the same also holds for backward merging. Before its application on node set ${\mathbf {N}}$ and their parents ${\mathbf {P}}$, the graph contains $|{\mathbf {N}}| \cdot |{\mathbf {P}}|$ edges between them. The resulting graph contains $|{\mathbf {P}}|$ edges from ${\mathbf {P}}$ to $N'$, and $|{\mathbf {N}}|$ edges from $N'$ to ${\mathbf {N}}$, much fewer than the ones in the initial graph. In addition, the total number of features contained in the graph is reduced by $(|{\mathbf {N}}|-1) \cdot |\mathbf {F'}|$. Note that application of those operations may result in a node containing no features. In this case the node can be removed, and edges from all its parents to all its children have to be added. Finally, OR merging always decreases the number of nodes and edges, while maintaining the total number of features. Specifically, for node set ${\mathbf {N}}$, the number of nodes is reduced by $|{\mathbf {N}}-1|$, and the edges are reduced by $(|{\mathbf {P}}| - 1) |{\mathbf {N}}|) + (|{\mathbf {C}}| - 1) |{\mathbf {N}}|)$, where ${\mathbf {P}}$ and ${\mathbf {C}}$ are the sets of parents and children of ${\mathbf {N}}$ respectively. Next, we will show that all operations are preserve the number of represented solutions.

#### Theorem 2

Application of Operation 1 and 2 does not affect the set of represented solutions by ${\mathcal {G}}$

#### Proof

Without loss of generality, we only prove the correctness of Operation 1. The correctness of Operation 2 can be shown following the same reasoning.

To prove correctness, it suffices to show that all paths into any node in ${\mathbf {P}}$ and out of any node $N_i$ contain the same set of features. Let $P_j$ be some parent of $N_i$. Initially, the set of features on the path through $P_j$ and $N_i$ are $var[P_j] \cup var[N_i]$. After performing the forward merging operation, the set of represented features is not affected, as $var[P_j] \cup var[N'] \cup (var[N_i] \setminus var[N']) = var[P_j] \cup var[N_i]$. Since the set of incoming edges of each $P_j$ as well as the set outgoing edges of each $N_i$ do not change, the set of represented solutions of the initial DAG is not altered. $\square$

A proof sketch for the OR merging operation follows. Observe that OR merging basically only groups some nodes together into one “super-node”. Based on this observation, it can easily be shown that it does not alter the solutions represented by the graph, as nodes are merged if and only if they have the same parents and children. Furthermore, this operation can be applied locally and independently to any part of the graph in any order, without affecting the final outcome.

Next, we will present algorithms that perform the forward and backward compression steps, using the respective merging operations. We do not provide any algorithm for the OR compression, as it simply is repeated application of OR merging until no more nodes can be merged.

### Algorithms for forward and backward compression

The forward compression starts from the root node *s* and separates its children into groups as follows. First it identifies the feature with the most occurrences among all its children and groups together all children that contain that feature (function $\textsc {SplitChildrenByFeatures}$ in Algorithm 6). This is repeated for all children that have not been grouped yet, until none remains. Next, after all children have been grouped, Operation 1 is performed on each such group $G_i$ (lines 3–9 in Algorithm 6). Application of Operation 1 is possible since all nodes in each group share common features and because all of them share the same parents, by construction. The aforementioned steps are repeated recursively for each newly created node $N'$ in place of *s*, until the leaf node *t* is reached. The procedure is summarized in Algorithm 6.
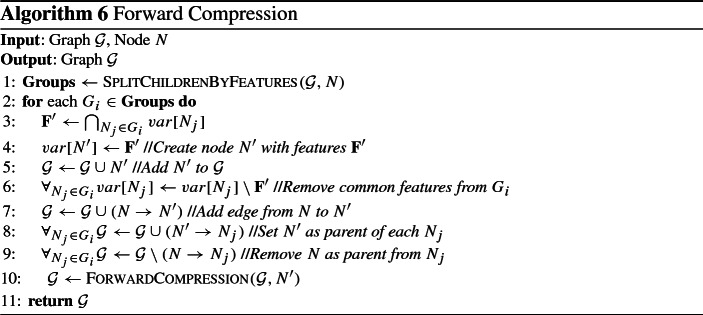

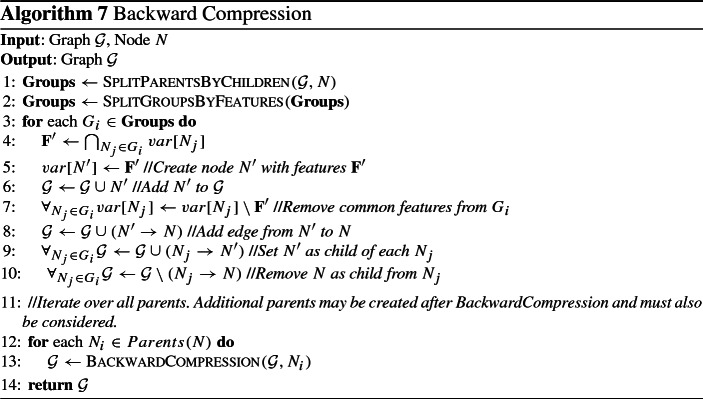


After completing the forward compression, an additional step is employed to further reduce the size of the DAG. This step is very similar to the forward compression, with a small modification. The backward compression starts from the leaf node *t* and groups all parents of *t* such that all nodes in the same group have the same children (function $\textsc {SplitParentsByChildren}$ in Algorithm 7). This is necessary in order to perform the backward operation. It was not required for the forward compression, as there it was guaranteed by construction that all nodes always have the same parents. Here however, it may happen that some parent of *t* is also a parent of some other parent of *t*, complicating things. Next, each such group is further split into sets of nodes that have common features, similarly to the forward step (function $\textsc {SplitsGroupsByFeatures}$ in Algorithm 7). Thus, after both splitting steps, nodes in each group have the same children and share features, allowing application of Operation 2 (lines 4–10 in Algorithm 7). Again, this procedure is applied recursively for all parents of *t*, *including the ones that are created after a recursive call of backward compression*,^7^ until the root node *s* is reached.

### Related methods

The problem of compactly representing feature sets is closely related to several other problems that have appeared in the computer science literature, which we briefly summarize and compare below.

**Binary decision diagrams** (BDDs) (Bryant 1986; Andersen 1997) are directed acyclic graphs that are used to compactly represent a Boolean function. Each node is associated with a Boolean feature and has two outgoing edges, one labeled “0” (or false) and one labeled “1” (or true), corresponding to the respective assignment of *x*. It has one root node, which is one of the Boolean features, and two leaf nodes “0” and “1”. Each path from the root node to one of the leaf nodes represents a feature assignment for the represented Boolean function. Depending on the leaf node, this assignment evaluates the represented Boolean function to true or false. **Ordered binary decision diagrams** (OBDDs) (Bryant 1986) are a special type of BDDs. They have the property that there is a unique structure for a given feature ordering, which is not necessarily the case for BDDs. The size of the OBDD highly depends on the feature ordering. The problem of finding the minimal OBDD is NP-complete (Bollig and Wegener 1996).^8^ There are various heuristics to find a good feature ordering; see (Rice and Kulhari 2008) for a survey on such methods. Another interesting type of BDDs are **zero-suppressed binary decision diagrams** (ZDDs) (Minato 1993). Often, especially when there are only a few solutions for a Binary function, ZDDs can be much smaller than OBDDs. It is straightforward to use BDDs to represent feature sets (which are a set of sets). Feature sets can be represented with Boolean functions by converting each set to an AND function and use an OR function between all such sets. For example, if ${\mathbf {M}} = \{ \{F_1, F_2\}, \{F_2, F_3, F_4\} \}$, the Boolean function $(F_1 \wedge F_2) \vee (F_2 \wedge F_3 \wedge F_4)$ represents all solutions in ${\mathbf {M}}$. In fact, OBDDs and ZDDs have already been used in this context (Minato 2001). The reason we chose not to use BDDs for our case is that they aren’t as easy to interpret, and it is harder to identify represented solutions visually. A path may contain “0” edges, which have to be filtered out in order to retrieve the respective feature set.

**Acyclic deterministic finite-state automata** (ADFA) (also known as directed acyclic word graphs (DAWG)) (Hopcroft et al. 2006; Revuz 1992; Daciuk et al. 2000) are used to represent a set of strings (called lexicon) in a compact way. ADFAs are directed acyclic graphs with nodes representing states and edges representing transitions between them. They contain one root and one leaf node, and each edge is associated with a letter. Each directed path from the root node to the leaf node represents a string, by concatenating the letters associated with each edge on that path. There are fast algorithms to incrementally construct a minimal size ADFA (Daciuk et al. 2000), or to minimize a given ADFA (Revuz 1992); see (Daciuk 2002) for a review and comparison of such methods. In our case, ADFAs could be used by converting each feature set to a string, and then using them to encode the whole set of feature sets. One way to do this is to choose a feature ordering, and to convert feature sets to strings by sorting them according to that ordering. This however is sub-optimal, as it unnecessarily restricts the resulting DAG to some feature ordering, which is not needed to actually represent feature sets. On the other hand, ADFAs also allow the repetition of letters, which is not needed in our problem as we deal with sets. Both of those reasons may potentially reduce their efficiency for compactly representing feature sets, which is why we decided to not use them.

We did not further investigate the possibility of using one of those data structures for our problem. We note that, due to their similarity with our proposed data structure, it may be that techniques used for minimization of BDDs or ADFAs could be applied in our case.

## Experimental evaluation

**Table 2:** Summary of the datasets used for the experimental evaluation

Dataset	#Samples	#Variables
Regression		
CnC Non-violent	2118	102
CnC Violent	1994	102
BlogData	60021	276
CT Slice	53,500	379
UJI Latitude	21,048	520
UJI Longitude	21,048	520
Classification		
Ada	4562	46
Musk	6598	166
Sylva	14,394	213
Madelon	2600	500
Gina	3468	970

We used 6 regression datasets and 5 binary classification datasets, with number of variables ranging from 46 to 970, and samples sizes between 1994 and 60,021

We evaluated TMFBS and compared it to the TIE* algorithm (Statnikov et al. 2013), the only alternative general algorithm for multiple solutions, with similar theoretical properties to TMFBS. For TIE* we used the IGS method for generating embedded datasets (see Sect. 6.3); we will refer to that specific TIE* instantiation as TIE*-IGS hereafter.

In our first experiment we compared (a) the number of solutions returned by each algorithm, as well as how many of them are statistically equivalent, (b) the predictive performance of the returned solutions,^9^ and (c) how the algorithms compare in terms of computational performance. Then, we investigated how the number of solutions and running time of both algorithms is affected by sample size. Finally, we show some examples of multiple solution graphs obtained on solutions returned by TMFBS.

**Data**. We considered binary classification and regression datasets. The data were collected from the UCI ML repository (Dietterich et al. 1994), using the following criteria: (a) they contain at least 1000 samples, to ensure that the equivalence tests have sufficient power, and (b) they contain at most 1000 features, so that all algorithms can terminate in a reasonable time frame. The datasets are shown in Table 2. More details about the data collection and pre-processing are given in “Appendix A”.

**Feature Selection Algorithms and Hyper-parameters**. For a fair comparison, we instantiated both TIE*-IGS and TMFBS with the FBS algorithm, as presented in Sect. 5. For continuous outcomes, we used the partial correlation test, and for binary outcomes we used a likelihood-ratio test based on logistic regression (see Sect. 2). For the significance level $\alpha$ of the conditional independence tests we considered 100 values uniformly spaced in the exponent of $10^{[-8, \ldots , \log _{10}(0.05)]}$, (i.e., the minimum $\alpha$ is $10^{-8}$ and the maximum is 0.05). A wide range of values for $\alpha$ is considered to allow for better tuning of FBS (see Sect. 4.3.2 for the motivation behind this).

**Predictive Modeling**. As predictive algorithms, we used ridge logistic and linear regression for binary classification and regression outcomes respectively.^10^ For the regularization parameter $\lambda$ of ridge regression we considered values $2^{[-30, \ldots , 30]}$, with a step size of 0.5 on the exponent (a total of 121 values).

**Equivalence Test for Solutions**. The goal of the experiments is to identify multiple, statistically equivalent Markov blankets. Following the recommendations given in Sect. 4.3, we combine a PEQ test with an IEQ test. Specifically, we (a) use a permutation-based variant of the variance test (Vuong 1989) for PEQ with 1000 permutations (see Sect. 4.3.4 for details), and (b) an IEQ test based on likelihood-ratio tests using logistic and linear regression models for classification and regression outcomes respectively. As sample sizes are relatively large, we set the significance level to 0.05 for both tests to minimize the number of false solutions.

**Analysis Protocol**. We employed a train/validation/test protocol, splitting the data to 60%/20%/20% respectively. As performance metrics we used the out-of-sample R$^2$ for regression and the area under the ROC curve for classification. Mean absolute error and root mean squared error for regression, as well as accuracy and balanced accuracy for classification have also been considered, and are presented in “Appendix B”. We used the following procedure: (a) on the training set, we trained a ridge regression model using the features identified by FBS for each combination of $\lambda$ and $\alpha$ (a total of $121 \cdot 100 = 12100$ combinations), (b) we selected the best combination based on its performance on the validation set, (c) we executed TMFBS and TIE*-IGS on the combined training and validation set using the best $\alpha$ and trained one model for each solution using the best $\lambda$, and (d) estimated their predictive performance on the test set.

**Implementations**. All algorithms were implemented by us in Matlab, except for ridge logistic regression, for which we used the implementation provided by the LIBLINEAR package (Fan et al. 2008).

### Evaluation of TMFBS and comparison with TIE*

**Table 3:** The table shows the summary of the comparison

Dataset	Speed-up	Perf.	Performance Range		#Sol. (#Eq.)	
			TMFBS	TIE*-IGS	TMFBS	TIE*-IGS
Regression						
CnC Non-violent	2.18	0.585	–	–	–	–
CnC Violent	2.31	0.588	[0.583, 0.590]	[0.583, 0.590]	2 (2)	2 (2)
BlogData	1.91	0.304	–	–	–	–
CT Slice	2.29	0.834	[0.834, 0.834]	[0.834, 0.834]	19 (16)	19 (16)
UJI Latitude	2.20	0.911	–	–	–	–
UJI Longitude	2.40	0.938	[0.938, 0.938]	[0.938, 0.938]	12 (9)	13 (10)
Classification						
Ada	1.97	0.904	–	–	–	–
Musk	2.24	0.991	–	–	–	–
Sylva	1.66	0.999	–	–	–	–
Madelon	1.81	0.646	–	–	–	–
Gina	2.09	0.932	[0.932, 0.934]	[0.932, 0.934]	1 (1)	1 (1)

It shows the speed-up of TMFBS over TIE*-IGS, the performance of the reference solution as well as the range of performances over all returned solutions, the number of additional solutions returned by each algorithm (#Sol.) (i.e., without counting the reference) and how many of them are statistically equivalent with the reference on the test set (#Eq.). Both algorithms produce very similar results in terms of the number of solutions and equivalent solutions identified, and all identified solutions have similar predictive performance. In terms of number of statistical tests performed, TMFBS performs around 2 times fewer tests than TIE*-IGS

For the first experiment we employed the aforementioned analysis protocol on the datasets shown in Table 2 datasets. In order to measure the speed-up of TMFBS over TIE*-IGS, we used the number of independence tests performed by each algorithm as a proxy of running time.^11^ For each solution, we compute the predictive performance obtained on the test set, as explained previously. Ideally, all identified equivalent solutions should have similar predictive performance. In order to verify that, we performed a test of performance equivalence for each identified solution with the reference solution on the test set. As a performance equivalence test we employed the permutation-based variance test, as described above. As the significance level is set to 0.05, we expect around 5% of equivalent solutions to be rejected on average. The results are summarized in Table 3.

First of all, we notice that both algorithms return a similar number of solutions. In fact, the solutions are identical, except for the UJI Longitude dataset, where TMFBS returned 12 solutions while TIE*-IGS returns 13. Those results agree with what we would expect from theory, as both algorithms have the same theoretical guarantees, although the results might differ in practice (see Sect. 6.3).

In terms of total number of returned solutions, we see that in most cases the algorithms identify only a single solution (7 out of 11 datasets), while in the rest the number of additional solutions is at most 19. Most of them are statistically equivalent, and even the ones that are not have very similar predictive performance (the difference is less than $0.1\%$ in all cases). The same also holds for other performance metrics; see Table 5 in “Appendix B” for additional results.

Even though the number of solutions is low, it is important* evidence that multiple solutions indeed exist in practice*. It is unlikely that those are false positives, given that the analysis is designed so that the number of false positive equivalences is minimized: we used large sample sizes, a relatively high threshold for the equivalence tests, filtered out solutions using a PEQ test, and used extensive tuning of the algorithms (see Sect. 4.3 for explanations of how the above affect the number of solutions). Furthermore, we there is no reason to believe a priori that the selected datasets do contain equivalent solutions, as the criteria used for selecting them are based on their size.

Finally, regarding speed-up, in all cases TMFBS is around 1.5–2.5 times faster than TIE*-IGS, showing that TMFBS is able to successfully take advantage of the search structure of FBS; larger speed-ups are expected with increasing number of features and solutions (see also the results of the next experiment, where speed-ups of 1–2 orders of magnitude are the norm).

### Number of solutions and speed-up with increasing sample size

Next, we performed an experiment to investigate how the number of solutions is affected by lower sample sizes, where more false positive solutions are expected due to lower power of the equivalence tests. Furthermore, we also check how the increased number of solutions affects the speed-up of TMFBS over TIE*-IGS. For this experiment, we only used the regression datasets, as the experiment is too time consuming for the classification datasets. The reason is that the logistic regression based test are significantly more computationally expensive than partial correlation tests, making such a large experiment infeasible.

We used the same experimental setup as before, but instead of using the full training set (i.e., the 60% of the original samples), we sampled $10\%$, $20\%$, $\ldots$, $90\%$ of the training data and used that as a training set to tune the hyper-parameters of FBS. The sampling was performed 20 times for each value, i.e., we performed a total of $20 \cdot 9 = 180$ runs of the analysis protocol for each dataset, and we report averages over the 20 runs. A limit of 1000 solutions was set, as in some small sample cases the TIE*-IGS algorithm would not terminate otherwise (the number of solutions often ranged in the millions).

**Fig. 5:**
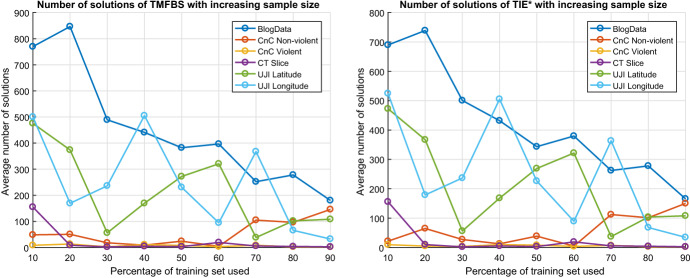
The figures show the average number of solutions over 20 runs with increasing sample size for TMFBS (left) and TIE*-IGS (right). First of all, we see that both algorithms return an almost identical number of solutions. We also see that the number of solutions tends to decrease with increasing sample size, as expected

Figure 5 shows how the number of solutions identified by TMFBS and TIE*-IGS varies with sample size. As before, the results are very similar for both algorithms. First we notice that, as expected, *the number of solutions tends to decrease with increasing sample size*. The only exception is for the CnC Non-violent dataset, where the number of solutions increases a bit for 70% of the samples or higher. Furthermore, for some cases (e.g., 40 and 70% for the UJI Longitude dataset) the number of solutions increases temporarily, and decreases afterwards. We were not able to identify the cause of this, but believe it may be an artifact of the experimental setup. Specifically, we believe it is due a combination of the relatively small number of runs (we used only 20 repetitions, due to the large computational cost) and the limit of 1000 solutions (again, to reduce the total computational cost). In any case, even though the number of solutions is not strictly monotonically decreasing with sample size, overall there is a clear monotonic trend for most datasets.

**Fig. 6:**
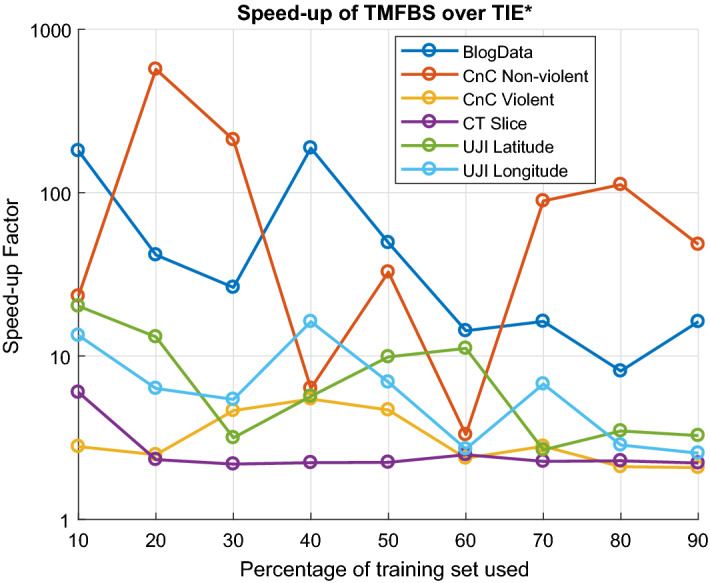
The figure shows the speed-up of TMFBS over TIE*-IGS with increasing sample size. We can see that TMFBS is typically 1–2 orders of magnitude faster than TMFBS on average

Figure 6 shows the speed-up of TMFBS over TIE*-IGS, computed as the ratio of statistical tests performed by TIE*-IGS over TMFBS, and averaged over all runs. We see that, on average, TMFBS significantly outperforms TIE*-IGS, typically being 1-2 orders of magnitude faster. The largest speed-ups are observed for lower sample sizes, where the number of identified solutions is also larger. Thus, the more solutions are identified, the larger the speed-up of TMFBS over TIE*-IGS is. Recall however that we set an upper limit of 1000 solutions for each run and, given that TIE*-IGS requires more tests per solution, we expect the speed-up to be even larger if no limit is enforced. Those results were expected, and can be explained by the search strategy of TMFBS which efficiently reuses computations, in contrast to TIE*-IGS which has to restart the search for each candidate solution.

### Multiple solutions graphs

**Fig. 7:**
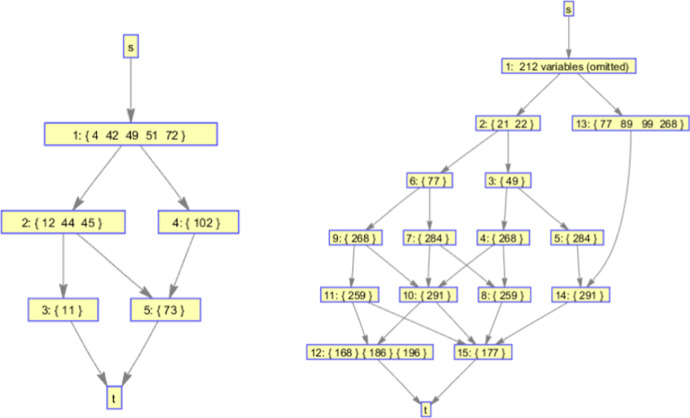
Multiple solutions graphs for the solutions on the CnC violent (left) and CT slice (right) datasets. The graphs contain 3 and 20 solutions, and require only 5 and 15 nodes respectively to represent them (excluding *s* and *t*). The first node contains 5 and 212 variables respectively, which correspond to variables that are contained in all solutions, i.e., variables that are indispensable

We show two multiple solution graphs (constructed using Algorithm 5) on the solutions of first experiment for the CnC violent and CT slice datasets in Fig. 7. The number of solutions are 3 and 20 for the CnC violent and CT slice datasets respectively. We see that the multiple solution graphs are able to efficiently encode all solutions, requiring only 5 and 15 nodes respectively. They also allow us to quickly identify interesting patterns. Recall that a solution can be read-off the graph by taking the union of features present in a path from *s* to *t*. Thus, the first node, which in both cases contains most of the features, corresponds to indispensable features. An example of replaceable features can be seen for the CnC dataset (graph on the left), where features 11 and 73 can be interchanged in all solutions. Another example can be seen on node 12 (graph on the right), which contains three sets of features (features 168, 186 and 196), which are replaceable for all solutions obtained by paths passing through node 12 (e.g., $1 \rightarrow 2 \rightarrow 6 \rightarrow 9 \rightarrow 11 \rightarrow 12$).

## Conclusion

We presented a novel strategy for extending feature selection algorithms to identify multiple statistically equivalent solutions, and proved under which conditions the algorithm is able to identify all solutions. Furthermore, we extended the taxonomy of features proposed by John et al. (1994) to also take multiplicity into account. We also proposed three definitions of statistical equivalence of solutions, as well as methods for testing them. In experiments, we showed that the proposed algorithm is significantly faster than the TIE* algorithm (Statnikov et al. 2013), the only other method with the same theoretical guarantees, while returning similar solutions. This happens because our algorithm directly takes advantage of the computations performed during the search, while TIE* does not.

As presented, the strategy can be used to extend greedy algorithms that consist of a forward and a backward phase. However, similar ideas could be used for a more general class of algorithms, namely methods that search in the space of solutions by adding or removing one or multiple features at each iteration. This would allow the extension of methods like recursive feature elimination, lasso (Tibshirani 1996), and stepwise selection (Kutner et al. 2004; Weisberg 2005) methods, to name a few.

A limitation of the experimental evaluation is that the algorithms have only been evaluated on regression and binary classification tasks. A more extensive evaluation, including larger numbers of datasets and other common tasks, such as multiclass classification, survival analysis and time course analysis, would be a valuable future research direction.

## A: Dataset collection and preparation for the evaluation of TMFBS

**Table 4:** Binary classification and regression datasets used in the experimental evaluation

Dataset	*n*	*p*	Outcome	Domain	Source
Ada	4562	46	Binary	Census Data	WCCI 2006 Challenge Guyon et al. (2006)
Musk (v2)	6598	166	Binary	Musk ActivityPrediction	UCI ML Repository Dietterich et al. (1994)
Sylva	14,394	216	Binary	Forest CoverTypes	WCCI 2006 Challenge Guyon et al. (2006)
Madelon	2600	500	Binary	Artificial	NIPS 2003 Challenge Guyon et al. (2004)
Gina	3568	970	Binary	HW DigitRecognition	WCCI 2006 Challenge Guyon et al. (2006)
Communities&Crime—Violent	1994	102	Real	CrimePrediction	UCI ML Repository Redmond and Highley (2010)
Communities&Crime—Non-violent	2118	102	Real	CrimePrediction	UCI ML Repository Redmond and Highley (2010)
BlogData	60,021	276	Real	Comments onBlog Posts	UCI ML Repository Buza (2014)
CT Slice	53,500	379	Real	CT SliceLocalization	UCI ML Repository Graf et al. (2011)
UJI IndoorLoc Latitude	21,048	520	Real	IndoorLocalization	UCI ML Repository Tsamardinos et al. (2014)
UJI IndoorLoc Longitude	21,048	520	Real	Indoo Localization	UCI ML Repository Tsamardinos et al. (2014)

*n* is the number of samples and *p* the number of features

Table 4 shows the list of all datasets, containing information about their number of samples *n* and features *p*, the type of features and outcomes, domain and sources.^12^ For all datasets, we performed the following pre-processing steps: (a) we removed all constant features as they do not provide any information about the outcome, (b) we removed features related to sample ids and other types of metadata, and (c) we removed samples containing missing values. Missing values were only present in a few datasets, and only for a few samples. In a real-world application, this should be handled appropriately to avoid biased results, using either missing value imputation or predictive algorithms that can handle missingness. However, for our purposes it would only unnecessarily complicate the tuning procedure and is not that important given the rare presence of missing values.

The communities & crime data contain a total of 18 continuous outcome features. Out of those, 8 measure the total number of committed crimes, 8 measure the number of crimes per 100K population, and 2 measure the number of violent and non-violent crimes per 100K population (by appropriately summing over the other outcomes). We chose to create two datasets, one measuring the violent and one the non-violent crimes. Note that, the number of samples differs, as there were missing values in the outcome features.

The UJIIndoorLoc datasets also contain multiple outcome values, such as the location (longitude and latitude), the floor of the building, the building ID and others. Again, we chose to create two datasets, one using the latitude outcome and one for the longitude.

The CT Slice data consist of samples obtained from CT scans on 97 patients. In this case, the training/validation/test splits where performed on the patients, and not directly on the samples (i.e., stratified on the patient id). This was done to avoid having data from the same patients both on the training and on the held-out data.

## B: Additional results for the comparison of TMFBS with TIE*

**Table 5:** The table shows additional performance metrics for the experiment in Sect. 8.1

Dataset	Metric	Reference	Range
Regression			
CnC Non-violent	R$^2$	0.585	–
	MAE	1303	–
	RMSE	1841	–
CnC Violent	R$^2$	0.588	[0.583, 0.590]
	MAE	254.0	[252.3, 255.4]
	RMSE	360.1	[359.0, 362.2]
BlogData	R$^2$	0.304	–
	MAE	9.27	–
	RMSE	32.54	–
CT Slice	R$^2$	0.834	[0.834, 0.834]
	MAE	7.56	[7.56, 7.56]
	RMSE	9.96	[9.96, 9.96]
UJI Latitude	R$^2$	0.911	–
	MAE	14.91	–
	RMSE	20.29	–
UJI Longitude	R$^2$	0.938	[0.938, 0.938]
	MAE	22.95	[22.93, 23.02]
	RMSE	30.91	[30.89, 30.98]
Classification			
Ada	AUC	0.904	–
	ACC	0.85	–
	BACC	0.762	–
Musk	AUC	0.991	–
	ACC	0.95	–
	BACC	0.946	–
Sylva	AUC	0.999	–
	ACC	0.992	–
	BACC	0.982	–
Madelon	AUC	0.646	–
	ACC	0.633	–
	BACC	0.633	–
Gina	AUC	0.932	[0.932, 0.934]
	ACC	0.862	[0.862, 0.863]
	BACC	0.861	[0.861, 0.862]

The performance of the reference solution is shown separately, while a performance range is shown over all equivalent solutions (if multiple). The table shows the R$^2$, mean absolute error (MAE) and root mean squared error (RMSE) for regression, and the area under the ROC curve, accuracy (ACC) and balanced accuracy (BACC) for classification. As the performance metrics are identical for TMFBS and TIE*, results are shown only once. We see that, the performance of all equivalent solutions is very close to the reference solution, regardless of task, dataset and metric

## Footnotes

1 This definition is essentially identical to the one given in (Lemeire 2007; Statnikov et al. 2013). Their definition requires also that $T \not \perp {\mathbf {X}}$ and $T \not \perp {\mathbf {Y}}$ hold which always holds in the context of feature selection, and thus can be dropped.

2 Extensions for other loss functions also exist (Golden 2003; Shi 2015).

3 For algorithms using conditional independence tests, such as forward–backward selection, the set of hyper-parameters contains the hyper-parameters of the algorithm (e.g., the significance threshold and the conditional independence test), as well as the hyper-parameters of the conditional independence test. While linear tests such as ones based on linear or logistic regression do not have any hyper-parameters to tune, other tests do have hyper-parameters. One such example is the kernel width of kernel-based conditional independence tests (Zhang et al. 2011).

4 This assumption is basically identical to admissibility rule I made by TIE* (see Figure 7 in Statnikov et al. (2013))

5 The proof is for IAMB (Tsamardinos et al. 2003b), which is FBS with a different, but provably correct, backward phase.

6 TIE* also assumes that ${\mathcal {Y}}$ generates embedded datasets in a way allowing all solutions to be identified.

7 In an actually implementation of Algorithm 7, the set of parents of *N* at line 12 has to be updated after each recursive call in the loop, as it may change during the loop.

8 Specifically, the decision version of the problem is NP-complete

9 We note that it is not the goal of this comparison to evaluate the performance of the single feature selection algorithm used by TMFBS and TIE*-IGS. Stepwise selection algorithms, such as the forward–backward selection algorithm used in the experiments, have been compared numerous times to other types of algorithms and have been shown to be competitive. See (Hastie et al. 2017; Borboudakis and Tsamardinos 2019; Tsamardinos et al. 2019) for recent comparisons of FBS and stepwise selection methods with lasso, Markov blanket discovery and information-theoretic algorithms.

10 We chose those models to compare equivalent solutions on an equal footing, as the conditional independence tests used by FBS only identify linear (or monotonic) dependencies. Using non-linear predictive models might obfuscate the results, as they would favor solutions which happen to identify variables that are non-linearly related to the outcome.

11 The number of tests is used instead of running time, as this is independent of the implementations used for the algorithms and tests. This is common practice when comparing algorithms based on independence tests (see (Aliferis et al. 2010) for example)
